# The acid-sensing ion channel 1a modulates anxiety- and depression-related behaviors via its influencing on the activity of corticotropin-releasing hormone-expressing neurons in the hypothalamic paraventricular nucleus in male mice

**DOI:** 10.1038/s41398-026-03946-2

**Published:** 2026-03-19

**Authors:** Jiayin Yue, Qilun Zhang, Mengyuan Wang, Xuelin Yao, Mengtian Wang, Ling Liu, Zhaohuan Huang, Yan Xing, Jinling Yan, Zihui Yan, Xing-Lei Song, Wei Wang

**Affiliations:** 1 Department of Endocrinology and Metabolism, Centre for Leading Medicine and Advanced Technologies of IHM, The First Affiliated Hospital of USTC, Division of Life Sciences and Medicine, University of Science and Technology of China, Hefei, 230001 China; 2 Core Research Facility, The Third People’s Hospital of Bengbu Affiliated to Bengbu Medical University (Bengbu Central Hospital), Bengbu, 233000 China; 3 Center for Advanced Interdisciplinary Science and Biomedicine of IHM, Division of Life Sciences and Medicine, University of Science and Technology of China, Hefei, 230027 China; 4 School of Biomedical Engineering, University of Science and Technology of China, Hefei, 230027 China; 5 School of Information Science and Technology, University of Science and Technology of China, Hefei, 230027 China; 6 Department of Anatomy and Physiology, Shanghai Jiao Tong University School of Medicine, Shanghai, 201318 China

**Keywords:** Molecular neuroscience, Neuroscience

## Abstract

A variety of studies show the involvement of acid-sensing ion channel 1a (ASIC1a) in the modulation of stress, however, the precise underlying mechanisms remain unclear. In this study, we provided evidence that ASIC1a, the Ca^2+^-permeable cationic ion channel, was co-expressed with corticotropin-releasing hormone (CRH) in the hypothalamic paraventricular nucleus (PVN). Downregulation of ASIC1a in the PVN CRH neuron decreased the hypothalamic-pituitary-adrenal (HPA) axis activity, which further ameliorated anxiety- and depression-related behaviors by reducing CRH neuron activity. In vitro, activation of ASIC1a elevated the intracellular Ca^2+^ concentration and promoted the expression of CRH by activating Ca^2+^/CaMKII/c-Fos signaling pathways. This study reveals a novel mechanism of the modulation of negative mood by ASIC1a and suggests a potential novel therapeutic target for stress-related diseases.

## Introduction

The stress, referring to a series of physiological and psychological reactions in response to external environmental changes or internal conflicts that threaten the homeodynamic equilibrium, is tightly controlled by neural systems that effectively switch vital physiological functions between homeostatic operation and emergency response. When an individual experiences extreme stressors that are out of their control, its stress response gets activated, which could lead to agitation and eventually stress-related disorders such as anxiety, depression, diabetes and hypertension [1–4].

The hypothalamic-pituitary-adrenal (HPA) axis is crucial for maintaining internal homeostasis through its modulation of stress in the central nervous system. It has been well documented to be the major component of the neuroendocrine network responding to both acute and chronic stress [2, 5, 6]. The corticotropin-releasing hormone (CRH) neuron in the paraventricular nucleus (PVN) of the hypothalamus controls HPA axis activity through initiating a hormonal cascade. Stress activates the CRH neuron, which results in the production and release of CRH into the pituitary, subsequently promoting the elevation of serum ACTH and corticosterone levels. Moreover, CRH neurons in the PVN (CRH^PVN^ neurons) receive and send projections to neighboring and extra-hypothalamic regions, modulating the activities of the autonomic nervous system and other stress circuits involved in controlling stress [7–10]. Therefore, the CRH^PVN^ neuron is a key player in orchestrating stress response [2, 8, 11].

Neuronal activities of CRH^PVN^ neurons are determined by ion channels and receptors expressed on the cell membrane of these neurons such as NMDA receptor [12], GABA_A_ receptor [13], AMPA receptor [3, 14] and voltage-gated potassium channel [15, 16]. Emerging evidence suggest that newly identifying ion channels and receptors are involved in precise mechanisms governing the elevated CRH^PVN^ neuronal activity in response to various stressors. Acid-sensing ion channels (ASICs), members of the epithelial Na^+^ channel/degenerin (ENaC/DEG) family, are proton-gated cationic channels. ASIC family consists of six subtypes including ASIC1a, 1b, 2a, 2b, 3, and 4 that are encoded by four genes. ASIC1a has a unique feature of Ca^2+^-permeability compared to the other ASIC subtypes. The activation of ASIC1a leads to increased membrane excitability and Ca^2+^ influx, which contributes to neuronal plasticity [17, 18]. Moreover, ASIC1a is associated with a variety of intracellular signaling molecules such as cAMP/PKA [19], Ca^2+^/CaMKII [20–22], NF-κB [23–26] and c-Fos [27]. These signaling molecules have been demonstrated to regulate CRH gene transcription in the PVN [28–30] and are involved in stress modulation [29].

Notably, ASIC1a is proven to be expressed in brain regions associated with stress modulation including amygdala, hippocampus, bed nucleus of the stria terminalis, nucleus accumbens and PVN [18, 31]. A variety of studies have demonstrated the involvement of ASIC1a in stress-related behaviors. Downregulation and inhibition of ASIC1a in the amygdala produce antidepressant-like effects [32]. Pharmacological inhibition and genetic knockdown of ASIC1a in the central nucleus of amygdala has shown to ameliorate anxiety-related behaviors [33]. Moreover, ASIC1a increases inhibitory activity in the basolateral amygdala and decreases anxiety during estrus [34]. Targeting ASIC1a gene or acutely inhibiting ASIC1a suppresses fear and anxiety independent of conditioning [27]. Additionally, overexpression of ASIC1a in transgenic mice increases fear conditioning [35]. Our recent study has indicated the presence of ASIC1a in the PVN [36, 37] and inhibition of ASICs may act on the HPA axis to alleviate the depression-related behavior in the chronic stress-subjected rat [37]. Nevertheless, whether ASIC1a in the PVN are involved in the modulation of HPA axis and mood behaviors remains unknown. Therefore, the present study aimed to investigate the specific role of ASIC1a in the PVN in regulating HPA axis function and anxiety- and depression-related behaviors.

## Materials and methods

### Animals

The C57BL/6J and *Crh-Cre* male mice used in this study were 6 to 8 weeks old unless otherwise stated. The C57BL/6J mice were purchased from SiPeiFu (Cat#B205, Beijing, China), and *Crh-Cre* mice were gifts from Prof. Ji Liu. The animals were bred in the facility at 22 °C and 35–55% humidity. They were maintained under a 12-h light/dark cycle (lights on from 8:00 am to 8:00 pm) with water and food available ad libitum. In order to avoid changes caused by the estrus cycle, we only considered male mice in the experiments. Animals were randomly assigned to control and treatment groups. All investigators were blinded to group allocation during data collection and analysis, group size was selected based on published reports in the field.

### Virus injection

The PVN injection was performed using a stereotactic frame (RWD, Shenzhen, China) under anesthesia with an intraperitoneal injection of pentobarbital sodium (20 mg/kg, i.p.). A volume of 100 nL virus was injected through calibrated glass microelectrodes connected to an infusion pump (micro4, WPI, USA) at a rate of 30 nL/min. The injection coordinates for the PVN were as follows: antero-posterior (AP), −0.79 mm from bregma; medio-lateral (ML), ±0.26 mm from the midline; dorsal-ventral (DV), −4.55 mm from the brain surface. The pipette remained in the injection site for 5–10 min at the end of infusion to avoid virus overflow.

To label CRH neurons, rAAV-Ef1α-DIO-EGFP-WPRE-pA (AAV-DIO-GFP, AAV2/9, 1.95 × 10^12^ vg/mL, Cat# PT-0795, BrainVTA, Wuhan, China) was injected into the PVN. For local knockdown of ASIC1a in CRH neurons within the PVN, recombinant viruses rAAV-CMV-DIO-mCherry-U6-shRNA (ASIC1a) (AAV-shASIC1a, AAV2/9, 5.00 × 10^12^ vg/mL, BrainVTA, Wuhan, China) were used. The viruses rAAV-CMV-DIO-mCherry-U6-shRNA (scramble) (AAV-mCherry, AAV2/9, 5.20 × 10^12^ vg/mL, Cat# PT-2788, BrainVTA, Wuhan, China) were used as controls. The complete sequences of the constructs in the supplemental material in Supplementary Data 1. The viral expression was checked after sacrificing of animals, and data were collected only from animals with correct viral expression. Initially, eleven mice were used in the experiment, one was excluded due to incorrect viral expression location, therefore, data from ten mice were used for the analysis of the results.

### Immunohistochemistry and imaging

Mice were deeply anesthetized by intraperitoneal injection with pentobarbital sodium, then perfused with ice-cold 0.9% saline followed by 4% paraformaldehyde (PFA). The brains were removed and placed in 4% PFA in PBS at 4 °C overnight, and then incubated in 30% (w/v) sucrose until they sank. Coronal slices were cut using a cryostat (CM1860, Leica, Germany) to a thickness at 40 µm. For immunofluorescence staining, the sections were incubated with blocking buffer (0.4% Triton X-100 plus 5% goat serum in PBS) for 1 h at room temperature, and then they were treated with primary antibodies diluted with blocking solution, including anti-c-Fos (1:500, rabbit, Cat# ab190289, Abcam, Cambridge, UK), anti-ASIC1a (1:500, guinea pig, Cat# ASC-014-GP, Alomone Labs, Jerusalem, Israel) at 4 °C for 24 h. After three washes with PBS, the slices were incubated with the corresponding fluorophore-conjugated secondary antibodies (1:500, Invitrogen, MA, United States) for 1.5 h at room temperature. Finally, sections were washed 3 × 5 min with PBS followed by mounting on slides with 80% glycerol solution. All sections were photographed by confocal microscopy (LSM980, Zeiss).

### Cannula infusion

Mice were anesthetized with isoflurane during the infusion, and the drug PcTx1 (15 μM in ACSF, 100 nL per side, Peptide Institute, Osaka, Japan) was administered through a polyethylene tubing between the injector and control infusion pump, and ACSF (100 nL) was locally applied as control. For intracranial microinfusion, the guide cannula (internal diameter 0.35 mm, RWD) was generally implanted 0.2 mm higher than the target nucleus of the PVN in mice one week before behavioral tests to ensure sufficient recovery time. After surgery, mice were housed individually. The cannula location was checked after sacrificing of animals, and data were collected only from animals with proper placement. Initially, eight mice were used in the experiment, one was excluded due to incorrect cannula placement, therefore, data from seven mice were used for the analysis of the results.

### Behavioral tests

#### Open-field test (OFT)

A white behavior test box (40 × 40 × 40 cm, length × width × height) was virtually divided into a center field (center, 20 × 20 cm) and a periphery field. For each test, the mice were placed in the periphery, and the locomotion of the animal was recorded with a video camera for 6 min. The time spent in the center or peripheral area was subsequently measured offline using EthoVision XT software (Noldus). We cleaned the instrument with 75% ethanol after each test to eliminate the odor effect.

#### Elevate plus maze (EPM)

An apparatus consisting of a central platform (6 × 6 cm) and two open arms (30 × 6 cm) orthogonal to two closed arms (30 × 6 × 20 cm) was placed 100 cm above the floor. Each animal was placed on the central platform facing one of the open arms and allowed to freely explore the maze for 6 min. The mice movement trajectory was recorded from above with a video camera. The time spent in the open arms was recorded and calculated offline using EthoVision XT software (Noldus). We cleaned the instrument with 75% ethanol after each test to eliminate the odor effect.

#### Tail suspension test (TST)

The mice were suspended by their tails, approximately 33 cm above the floor. Each mouse was affixed to a hook positioned 1 cm from the tail tip using tape. A test period of 6 min was recorded, during which the duration of immobility was measured. For the TST, the results were calculated by researchers who were blind to the experiment. Immobility was defined as the absence of any observable movement except for whisker movement and respiration.

#### Forced swimming test (FST)

The mice were placed into a circular bucket (height, 25 cm; diameter, 10 cm), filled with water (15-cm depth) maintained at 24–26 °C. They were placed in the container with their backs to the wall and the front paws touching the water, and allowed to swim freely for 6 min. After each session, the mouse was dried with a towel and then returned to its home cage. For the FST, the results were calculated by researchers who were blind to the experiment. Each mouse was judged to be immobile when it ceased struggling and remained floating motionless in the water, making only those movements necessary to keep its head above water. For behavior tests, the data of the mice that did not move throughout the entire test were excluded. All behavioral data were included in the analysis, no data were excluded due to immobility.

### Hormone measurement

For the animal experiment, blood samples were collected via the ocular vein after 0.5 h of FST stimulation between 11:00 a.m. and 1:00 p.m. The blood was centrifuged (1500 g, 15 min) for plasma, and then stored at −80 °C for subsequent testing. A panel of hormones associated with stress was measured using ELISA kits for ACTH (Cat. D721184, BBI-life sciences) and corticosterone (Cat. D721183, BBI-life sciences). For the cell experiment, the release level of CRH was measured using ELISA kits for human (Cat# MM-1014H1, MEIMIAN, Jiangsu, China) and for mice (Cat# MM-0509M1, MEIMIAN, Jiangsu, China). The experiments were conducted according to the manufacturer’s instructions provided with the kit.

### Ca^2+^ imaging and fiber photometry

The virus rAAV-hSyn-DIO-GCaMP6s-WPREs (AAV-DIO-GCaMP6s, AAV2/9, 1.20 × 10^13^ vg/mL, 200 nL, Cat# PT-0091, BrainVTA, Wuhan, China) was unilaterally injected in the PVN of *Crh-Cre* mouse and allowed to express for 3 weeks. An optical fiber (250 μm diameter core, NA 0.37, Newdoon, Hangzhou, China) was placed on the PVN of *Crh-Cre* male mouse and connected to a fiber photometry system (ThinkerTech, Nanjing, China). The excitation light was generated by a 480-nm LED, reflected with a dichroic mirror, and delivered to the brain to excite the GCaMP6. The excitation light was passed through another band-pass filter, into a complementary metal-oxide semiconductor detector (DCC3240M, Thorlabs), and finally recorded by a LabVIEW program (SingleColorMultiChannel Acquisition Software, ThinkerTech, Nanjing, China).

Fiber photometry signals were processed with acustom-written MATLAB software, which is available at https://zenodo.org/record/6456623#.YlaF_8hByUk (Zenodo ID: 6456623; Zenodo DOI: 10.5281/zenodo.6456623; ThinkerTech Nanjing Bioscience, 2020). Briefly, all the data were segmented on the basis of behavioral events and baseline phase. The values of Ca transients change (ΔF/F) from −2 s to 6 s (0 s represents the onset of each stimulus), where ΔF/F = (F-F0)/F0 for each trial and F0 was defined as the average value in control time. For fiber photometry, data of the mice from loss of the optical fiber and incorrect optical fiber or viral expression were excluded. All of the data were included in the analysis, no data were excluded due to incorrect optical fiber.

### Cell culture

Human neuroblastoma cell line BE (2)-C was cultured in DMEM (VivaCell Biosciences, Shanghai, China) supplemented with 10% FBS (ExCell Bio, Suzhou, China) and 1% P/S (Biosharp, Hefei, China) at 37 °C, containing 5% CO_2_. Primary hypothalamic neurons were prepared as previously described [38]. At day 7 of neuronal culturing, lentiviral overexpression or knockdown of ASIC1a was introduced, and the cells were subsequently cultured for an additional 48 h for experimentation.

### Plasmid and lentiviral construction

For knockdown experiments, shRNA sequences were cloned into pGreenPuro hairpin lentivector (SBI, California, USA) separately, targeting human ASIC1a (5’-3’: CTATGGAAAGTGCTACACGTT) and mouse ASIC1a (5’-3’: CCTCAAGCATACGGAATCT). Overexpression vectors were generated by inserting human/mouse ASIC1a sequence into pLVX-Green1-N1 vector. All plasmids were constructed using restriction enzyme digestion (Yugong Biolabs, Jiangsu, China) and T4 ligation (Monad Biotech, Suzhou, China) methods, and were confirmed by Sanger sequencing. Lentiviral particles were produced by transient co-transfection of the aforementioned constructed plasmids along with pCMV-dR8.2 and pVSV-G into 293 T cells, followed by the collection of viral particles through ultracentrifugation.

### Western blot assay

The cells were lysed on ice using 1% Triton X-100 in PBS, followed by sonication and centrifugation at 13,400 g for 15 min at 4 °C. The supernatant was then subjected to SDS-PAGE and transferred onto nitrocellulose membranes (running buffer: 0.025 M Tris, 0.25 M Glycine, 0.1% SDS; transfer buffer: 25 mM Tris, 192 mM Glycine, 20% Methanol). After blocking (5% skimmed milk), the membranes were incubated with primary antibodies overnight, including that CRH/CRF Polyclonal antibody (Cat#10944-1-AP, Proteintech, Wuhan, China), ASIC1a Polyclonal antibody (Cat#27235-1-AP, Proteintech, Wuhan, China), c-Fos monoclonal antibody (Cat#66590-1-Ig, Proteintech, Wuhan, China), phospho-CaMKII alpha/delta (Thr286) antibody (Cat#AF3493, Affinity Biosciences, Jiangsu, China) and CaMKII α antibody (Cat#WL03453, Wanlei Bio, Shenyang, China), and β-actin antibody (Cat#WL01372, Wanlei Bio, Shenyang, China), and then followed by HRP-conjugated secondary antibodies (1:5000). ECL reagent (US Everbright) was added, and the bands were visualized using a chemiluminescence imager (Cat#SH-523, SHST, Hangzhou, China). The dilution of antibodies was conducted according to the manufacturer’s instructions.

### RNA extraction and qPCR

Total RNA was extracted using the Trizol reagent kit (Invitrogen, USA), followed by reverse transcription using a reverse transcription kit (Vazyme, Nanjing, China). RT-qPCR was conducted on the QuantStudio 3 platform. All experiments were conducted following the manufacturer’s instructions.

qPCR primer:

CRH F: 5’-3’ CAGCCCTTGAATTTCTTGCAG,

CRH R: 5’-3’ GACTTCTGTTGAGATTCCCCAG,

ASIC1a F: 5’-3’ GGAAGTGCCAGAAGGAGG,

ASIC1a R: 5’-3’ GCGGGATGGTGAGGTAGGAT,

c-Fos F: 5’-3’ CCAGCATGGGCTCTCCTGTCAACACA,

c-Fos R: 5’-3’ TCCGTAAGGATGGGGCGCTCTGGT,

β-actin F: 5’-3’ CATTGCTGACAGGATGCAGAAGG,

β-actin R: 5’-3’ TGCTGGAAGGTGGACAGTGAGG

### Neuron transfection and calcium imaging

Calcium phosphate transfection was performed in cultured hypothalamic neurons grown on confocal dishes on day in vitro (DIV) 7–10. Before transfection, the culture medium was replaced with fresh Neurobasal medium, and the original medium was retained. For each dish, 2 μg of GCaMP6 plasmid together with 2 μg other plasmids (vector, mCherry-ASIC1a, or shASIC1a) was diluted in 60 μL CaCl_2_ solution (0.3 M) with pipetting. An equal volume (60 μL) of HBSS (280 mM NaCl, 1.5 mM Na_2_HPO_4_, 50 mM HEPES, pH 6.9) was then added. The mixture was combined immediately by gentle pipetting and promptly transferred into the dish. After incubation at 37 °C for 1–1.5 h, the medium was replaced with CO_2_-saturated Neurobasal medium (washing medium) to remove excess calcium phosphate precipitates. Finally, the washing medium was replaced with a 1:1 mixture of the original conditioned medium and fresh Neurobasal medium supplemented with B27, and the dish was returned to the culture incubator.

Calcium imaging was conducted at DIV 14–16, in transfected neurons. Confocal dishes with transfected neurons were mounted in a standard extracellular solution and imaged consecutively using a Nikon A1R laser scanning confocal microscope with a 20 × objective. GCaMP6 was excited using a 488 nm laser, and mCherry (used to label transfected neurons) was excited with a 561 nm laser. Following a 30-s baseline recording at pH 7.4, the perfusate was rapidly switched to pH 6.5 for 20 s, followed by a return to pH 7.4 for an additional 60 s. GCaMP6 fluorescence change representing intracellular Ca^2+^ dynamics was calculated as F/F0, where F0 represents the median fluorescence intensity during the 30-s baseline period. For cell calcium imaging, low transfection efficiency were excluded. All of the data were included in the analysis, no data were excluded.

### Statistical analysis

The statistical analyses and graphing were performed using GraphPad Prism 8.0 (GraphPad Software, Inc., San Diego, CA, USA) or SPSS (version 18, IBM, Chicago, IL, USA). Normality of data was checked with the Shapiro-Wilk test and homoscedasticity was checked using Bartlett’s test. For statistical comparisons, a two-tailed unpaired t test was used to compare two groups. One- and two-way ANOVA tests followed by post hoc analyses were used to statistically analyze the data from the experimental groups with multiple comparisons. All experiments were repeated in at least three independent biological replicates. Statistical significance levels are indicated as *p < 0.05; **p < 0.01; ***p < 0.001, ****p < 0.0001; n.s. indicates not statistically significant. All data are expressed as the mean ± SEM. Potential outliers were assessed using Grubbs’ test, and no statistically significant outliers were identified.

## Results

### Morphological evidence of ASIC1a in the PVN, whose blockade reduced HPA axis activity and improved anxiety- and depressive-like behaviors

**Fig. 1:**
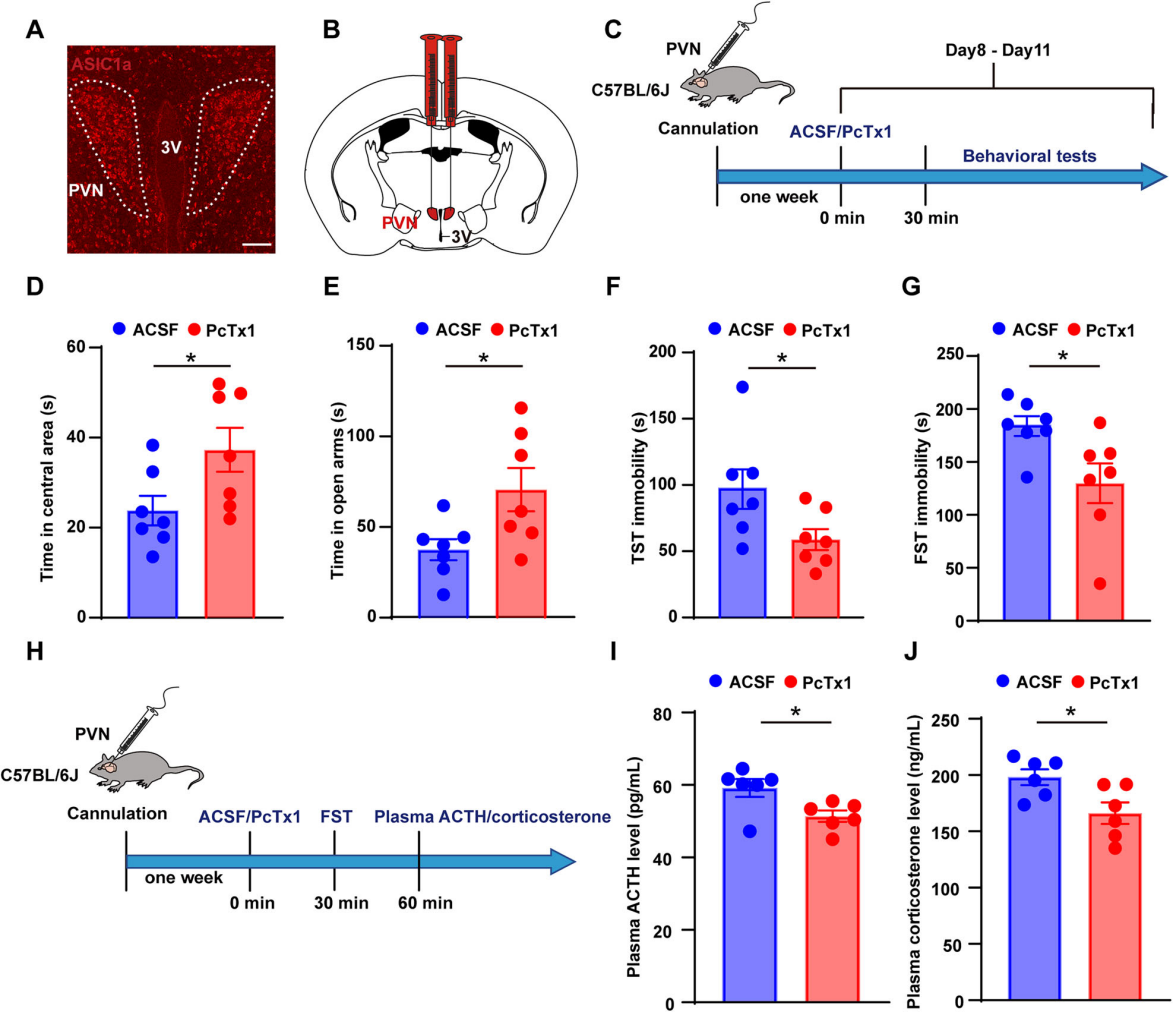
Inhibition of ASIC1a in the PVN ameliorated anxiety- and depression-like behaviors and decreased HPA axis activity. **A** Representative immunofluorescence image for ASIC1a (red) in the PVN from a naïve mouse. Scale bar, 100 μm. **B** Schematic of bilateral cannula injection sites into the paraventricular nucleus (PVN) (coordinates: anterior-posterior = –0.79, medial-lateral = ±0.26, dorsal-ventral = –4.35). **C** Schematic of pharmacological experimental procedure. **D** The bar graph illustrates the behavioral analysis, showing that the PcTx1 group exhibited a significant increase in the time spent in the center of the open-field test (OFT) compared to the ACSF group (n = 7 per group). *p < 0.05 (unpaired *t*-test). **E** The bar graph illustrates the behavioral analysis, showing that the PcTx1 group exhibited a significant increase in the time spent in the open arms of the elevated plus maze (EPM) compared to the ACSF group (n = 7 per group). *p < 0.05 (unpaired *t*-test). **F** The bar graph illustrates the results of the tail suspension test (TST), showing that the PcTx1 group exhibited a significant reduction in immobility time compared to the ACSF group (n = 7 per group). *p < 0.05 (unpaired t-test). **G** The bar graph illustrates the results of the forced swimming test (FST), showing that the PcTx1 group exhibited a significant reduction in immobility time compared to the ACSF group (n = 7 per group). *p < 0.05 (unpaired *t*-test). **H** Experimental paradigm for blood collection after forced swimming. **I** The bar graph illustrates plasma ACTH levels in the PcTx1 group and ACSF group following stress stimulation (n = 6 per group). *p < 0.05 (unpaired t-test). **J** The bar graph illustrates plasma corticosterone levels in the PcTx1 group and ACSF group following stress stimulation (n = 6 per group). *p < 0.05 (unpaired t-test). See also Supplementary Data 2.

Consistent with previous studies [36, 37], the expression of ASIC1a in the PVN was confirmed by immunofluorescence staining (Fig. 1A). The specificity of the antibodies used in this study was validated via immunofluorescence and Western blotting. No ASIC1a signal was detected in the brains of ASIC1a knockout mice (Supplementary Figure 1A), and Western blotting with an anti-ASIC1a antibody identified a single band at ~60 kD (Supplementary Figure 1B). In order to evaluate whether ASIC1a in the PVN modulates the stress response, PcTx1, a specific ASIC1a inhibitor, was bilaterally infused into the PVN through implanted catheters in naïve mice, followed by behavioral assessments. As a control, an identical procedure was performed using artificial cerebrospinal fluid(ACSF) as the vehicle solution (Fig. 1B, C and Supplementary Figure 1C). The PcTx1 group exhibited a significant increase in the time spent in the center of the open-field test (OFT) and the open arms of the elevated plus maze (EPM) compared to the ACSF group (Fig. 1D, E). However, no significant differences were observed between the two groups in terms of total distance traveled or average speed in either the OFT or EPM (Supplementary Figure 2A–D). In the tail suspension test (TST) and forced swimming test (FST), the PcTx1 group showed a significant reduction in immobility time (Fig. 1F, G). Additionally, plasma ACTH and corticosterone levels were significantly decreased in the PcTx1 group following acute stress challenges (Fig. 1H–J), while baseline plasma ACTH and corticosterone levels prior to stress showed no significant differences between the two groups (Supplementary Figure 2E, F). Finally, we observed a significant reduction in c-Fos expression specifically within ASIC1a-positive neurons in the PVN of PcTx1-treated mice compared to controls after FST stimulation (Supplementary Figure 2G, H).

### Knockdown of ASIC1a decreased the CRH^PVN^ neuronal activity and ameliorated anxiety- and depression-related behaviors

**Fig. 2:**
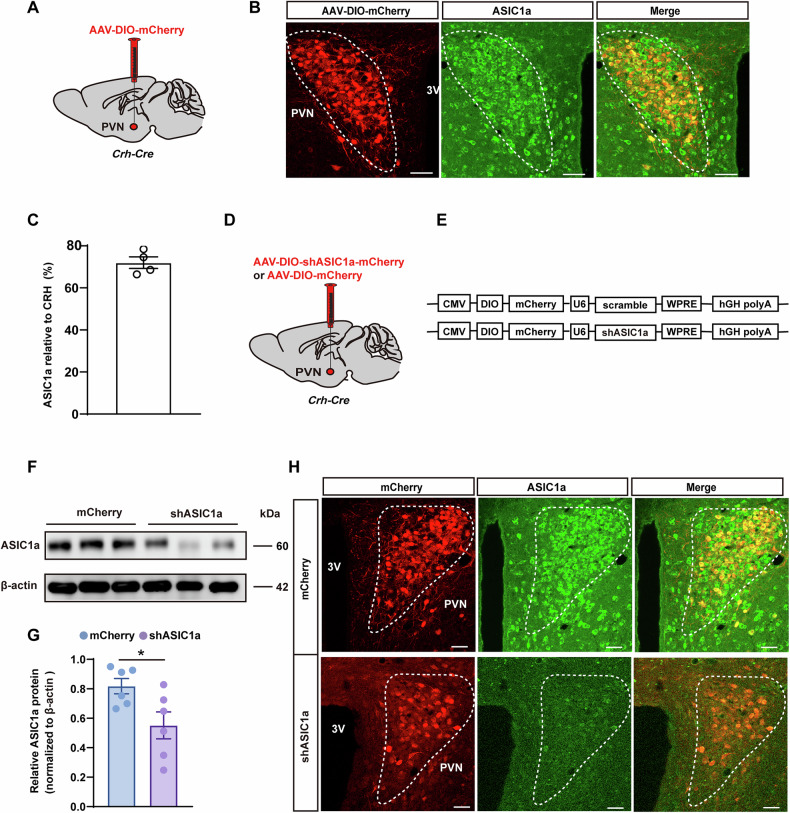
ASIC1a neurons in the PVN that co-localize with CRH neurons. **A** Experimental paradigm for viral injection of AAV-DIO-mCherry in *Crh-Cre* mice. **B,** **C** Representative images (**B**) and statistical data (**C**) showing ASIC1a (green) neurons in the PVN co-localized with CRH (red) neurons. Scale bars, 50 μm. **D** Experimental paradigm for viral injection of AAV-DIO-shRNA in *Crh-Cre* mice. **E** Schematic illustration of the construction strategy for ASIC1a knockdown. **F,** **G** Representative Western blot images (**F**) and quantification (**G**) demonstrating that AAV-DIO-shASIC1a injection reduced ASIC1a expression in the PVN (n = 6 per group). *p < 0.05 (unpaired t-test). **H** Immunofluorescence images showing mCherry-positive neurons co-labeled with ASIC1a in the AAV-mCherry and AAV-shASIC1a groups. Scale bars, 50 μm. See also Supplementary Data 2.

**Fig. 3:**
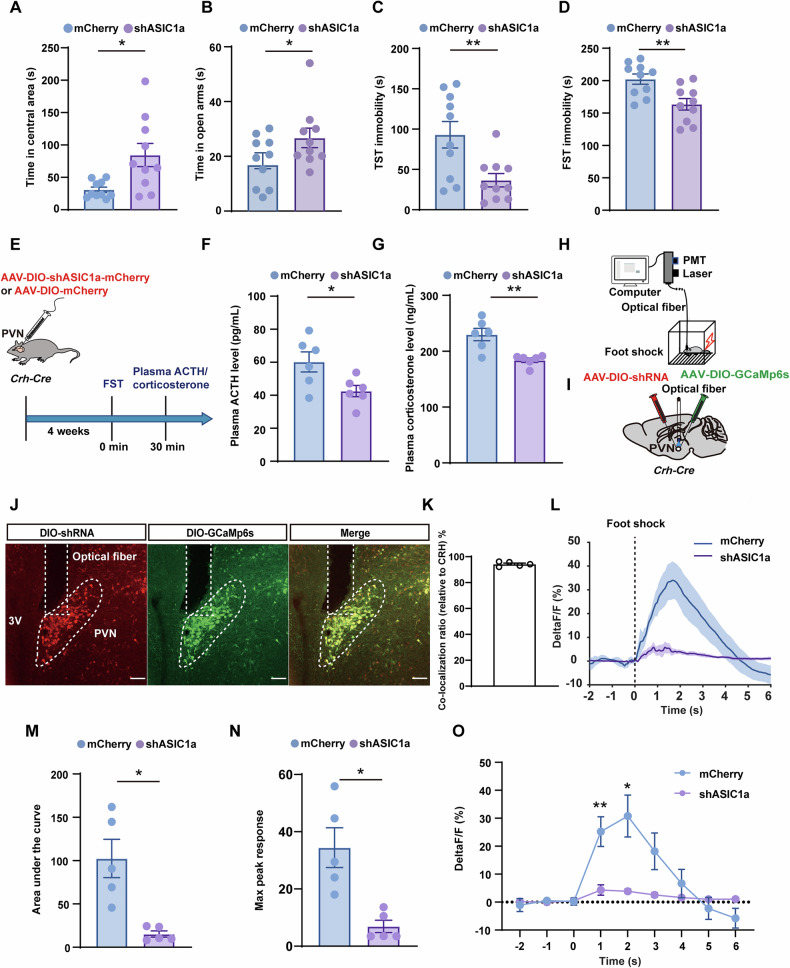
Knockdown of ASIC1a decreased the CRH^PVN^ neuronal activity and ameliorated anxiety- and depression-related behaviors. **A-D** Behavioral effects of the genetic knockdown of ASIC1a in CRH^PVN^ neurons. Figure 3A shows the open-field test (OFT), Fig. 3B shows the elevated plus maze (EPM), Fig. 3C shows the tail suspension test (TST), and Fig. 3D shows the forced swimming test (FST), demonstrating significant behavioral differences between the AAV-mCherry group and the AAV-shASIC1a group (n = 10 per group). *p < 0.05, **p < 0.01 (unpaired t-test). **E** Experimental paradigm for blood collection following forced swimming. **F,** **G** Plasma ACTH levels (**F**) and corticosterone levels (**G**) were significantly reduced in the AAV-shASIC1a group compared to the AAV-mCherry group after forced swimming (n = 6 per group). *p < 0.05, **p < 0.01 (unpaired t-test). **H** Schematic of the real-time optical fiber photometry assay. **I** Experimental paradigm for viral injection of AAV-shRNA and AAV-DIO-GCaMP6s in *Crh-Cre* mice. **J,** **K** Representative images (**J**) and statistical data (**K**) showing AAV-shRNA and AAV-DIO-GCaMP6s expression in the PVN. Scale bars, 50 μm. **L-O** Calcium activity analysis of CRH^PVN^ neurons in the AAV-shASIC1a group compared to the AAV-mCherry group. Figure 3L shows average calcium activity, Fig. 3M shows the area under the curve, Fig. 3N shows the maximum peak value, and Fig. 3O shows calcium signals at specific time points (n = 5 per group). *p < 0.05, **p < 0.01 (unpaired t-test and two-way ANOVA). See also Supplementary Data 2.

Since CRH^PVN^ neurons serve pivotal roles in the modulation of stress, we first examined whether these neurons express ASIC1a. By injecting AAV-DIO-mCherry into the PVN of *Crh-Cre mice* (Fig. 2A), immunofluorescence staining showed that ASIC1a was highly co-labeled with the mCherry + CRH^PVN^ neurons (Fig. 2B, C). To further explore the role of ASIC1a in the CRH^PVN^ neurons, we designed a Cre-dependent AAV vector expressing ASIC1a-targeting short-hairpin RNAs (shRNAs) for knockdown experiments. Two recombinant adeno-associated viruses (rAAVs) were used in *Crh-Cre* mice: rAAV-CMV-DIO-mCherry-U6-shRNA (ASIC1a) (AAV-shASIC1a) and rAAV-CMV-DIO-mCherry-U6-shRNA (scramble) (AAV-mCherry) as a control (Fig. 2D, E). The AAV-shASIC1a construct utilized the U6 promoter to drive ASIC1a-specific shRNA expression, leading to post-transcriptional silencing of ASIC1a. The CMV-DIO-mCherry cassette ensured Cre-dependent expression of the fluorescent reporter mCherry, allowing visualization of transfected neurons and confirmation of ASIC1a knockdown specificity. In contrast, the AAV-mCherry construct expressed a scrambled shRNA sequence under the U6 promoter, serving as a negative control without altering ASIC1a expression. Four weeks after virus injection PVN, Western blot analysis demonstrated a significant reduction in ASIC1a protein levels in the AAV-shASIC1a group compared to the AAV-mCherry group (Fig. 2F, G). Immunofluorescence staining further confirmed reduced ASIC1a expression in the AAV-shASIC1a group (Fig. 2H). Behavior tests showed that CRH^PVN^ neuron-specific ASIC1a knockdown led to increased times in the central area of the OFT and the open arms of the EPM (Fig. 3A, B). No significant differences were observed between groups in total distance traveled or average speed in the OFT and EPM (Supplementary Figure 3A–D). Additionally, mice with ASIC1a knockdown exhibited significantly reduced immobility time in the TST and FST compared to controls (Fig. 3C, D). Plasma ACTH and corticosterone levels were also significantly decreased in the AAV-shASIC1a group following acute stress challenges (Fig. 3E–G), while baseline levels prior to stress showed no significant differences between the two groups (Supplementary Figure 3E, F).

To examine the effects of ASIC1a on the activity of CRH^PVN^ neurons, fiber photometry recordings were performed in mice injected with virally expressed fluorescent Ca^2+^ indicator GCaMP6s (AAV-DIO-GCaMP6s) mixed with either AAV-shASIC1a or AAV-mCherry into the PVN. A foot shock was applied to activate CRH^PVN^ neurons (Fig. 3H, I). Co-localization of red fluorescent protein with green fluorescence confirmed successful targeting of CRH^PVN^ neurons (Fig. 3J, K). In response to foot shock, bulk Ca^2+^ signals (ΔF/F) in the AAV-mCherry group showed overall > 30% change, whereas the AAV-shASIC1a group exhibited about 10% changes (Fig. 3L). Analysis of the area under the curve, peak amplitude, and specific time points revealed statistically significant differences between the two groups (Fig. 3M–O), indicating that ASIC1a knockdown attenuates neuronal activity in response to acute stress. Similarly, during the FST, bulk Ca^2+^ signals in the AAV-shASIC1a group were decreased compared to the AAV-mCherry group (Supplementary Figure 4A, B), with reduced area under the curve and peak amplitude specifically associated with struggling behavior (Supplementary Figure 4C, D). Consistent with these findings, c-Fos analysis after forced swimming showed a significant reduction in c-Fos expression within the mCherry-positive cells in the AAV-shASIC1a group, with no significant change in the mCherry-negative cells between two groups (Supplementary Figure 4E–G). Western blot analysis demonstrated a significant reduction in CRH protein levels in the PVN of AAV-shASIC1a group compared to the AAV-mCherry group following exposure to the forced swim test (Supplementary Figure 4H, I).

### ASIC1a modulated CRH expression and secretion

**Fig. 4:**
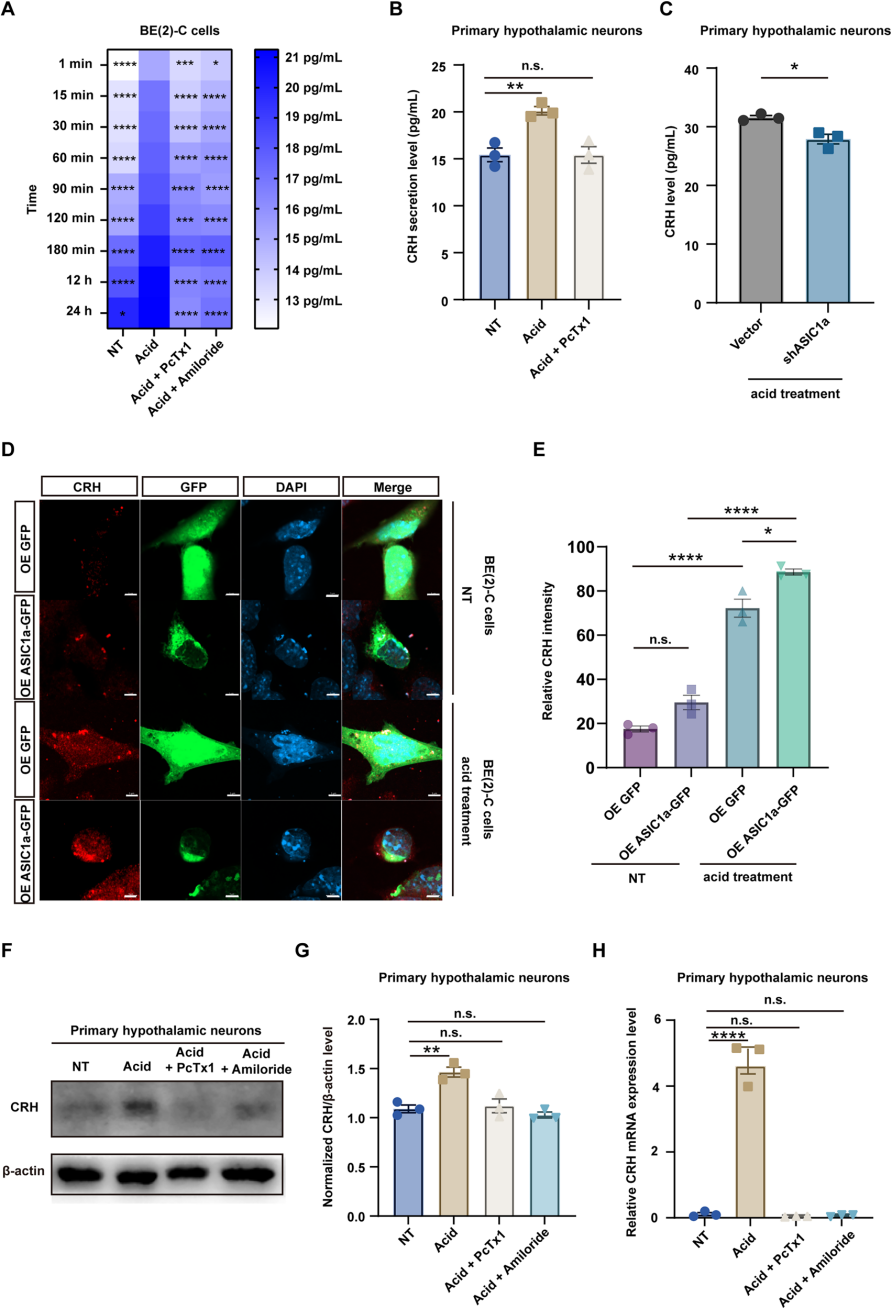
ASIC1a modulated CRH expression and secretion. **A** The heatmap shows the release levels of CRH in BE (2)-C cell culture supernatants detected by ELISA. The BE (2)-C cells were treated with HBSS with a pH of 6.5 for 5 min or pretreated with 100 nM PcTx1 or 200 nM amiloride for 5 min and maintained in HBSS pH 6.5. The supernatants were collected at 1, 15, 30, 60, 90, 120, 180 min, 12 h, and 24 h for detection. The experiment was conducted with three independent biological replicates, ensuring the presence of the drug throughout both the culture and acid treatment stages (n = 3 per group). *p < 0.05, ***p < 0.001, ****p < 0.0001 (two-way ANOVA). **B** The bar graph shows CRH release levels in neuronal culture supernatants detected by ELISA. The experimental treatments were conducted as in Fig. 4A, and supernatants were collected at 3-h time point for detection (n = 3 per group). **p < 0.01 (one-way ANOVA). **C** The bar graph shows CRH levels in the supernatant of ASIC1a knockdown neuronal culture medium detected by ELISA after HBSS pH 6.5 treatment and the supernatants were collected at 3-h time point for detection (n = 3 per group). *p < 0.05 (unpaired *t-*test). **D** Representative confocal images (scale bar = 5 μm) of CRH in BE (2)-C cells overexpressing GFP/ASIC1a-GFP for 48 h, before or 30 min after treatment with HBSS at pH 6.5. **E** The bar graph illustrates the relative fluorescence intensity of CRH shown in Fig. 4D (n = 3 per group). *p < 0.05, ****p < 0.0001 (one-way ANOVA). **F** Representative western blot assay showing the protein expression levels of CRH in primary hypothalamic neurons. Experimental treatments were conducted as in Fig. 4A, and the cells were detected at the 3-h time point. **G** The bar graph illustrates the grayscale scanning analysis of Fig. 4F, showing the relative expression levels of CRH (n = 3 per group). **p < 0.01 (one-way ANOVA). **H** The bar graph shows the mRNA expression levels of CRH in primary hypothalamic neurons detected by qPCR. Experimental treatments were conducted as in Fig. 4F (n = 3 per group). ****p < 0.0001 (one-way ANOVA). See also Supplementary Data 2.

To investigate the mechanisms underlying the effects of ASIC1a on CRH expression and release, the neuroblastoma cell line BE (2)-C cells were treated with Hanks’ balanced salt solution (HBSS) at pH 6.5 [22, 39–43] for 5 min [44], followed by a return to normal serum-free culture medium. This acid treatment significantly enhanced CRH secretion, with a notable peak at 60 min post-acid treatment, where the mean CRH concentration surged from 13.9 pg/mL to 17.79 pg/mL. In contrast, the administration of ASIC1a blockers PcTx1 or amiloride attenuated this response, moderating the mean CRH concentrations to 15.13 pg/mL and 15.29 pg/mL, respectively. Quantitative assessments were performed at intervals spanning from 1 min to 24 h following the treatment (Fig. 4A). This finding was further validated in primary cultured hypothalamic neurons. Following a 5-minute acid treatment and a subsequent 3-hour post-acid incubation, the mean concentration of CRH secreted by neurons increased from 15.43 pg/mL to 20.12 pg/mL. In contrast, the PcTx1 treatment group maintained a stable CRH level of 15.42 pg/mL (Fig. 4B). ASIC1a knockdown also significantly attenuated the acid-induced increase in CRH release, with the mean CRH secretion level reduced from 31.58 pg/mL to 27.88 pg/mL 3 h after acid treatment in primary neurons (Fig. 4C). Immunofluorescence staining demonstrated a significant upregulation of CRH expression in BE (2)-C cells treated with acidic HBSS for 30 min where ASIC1a was overexpressed (Fig. 4D, E), and this effect was inhibited by both PcTx1 and amiloride in cultured hypothalamic neurons at both protein and mRNA levels (Fig. 4F–H).

### ASIC1a facilitated CRH transcription via Ca^2+^/CaMKII/c-Fos pathway

**Fig. 5:**
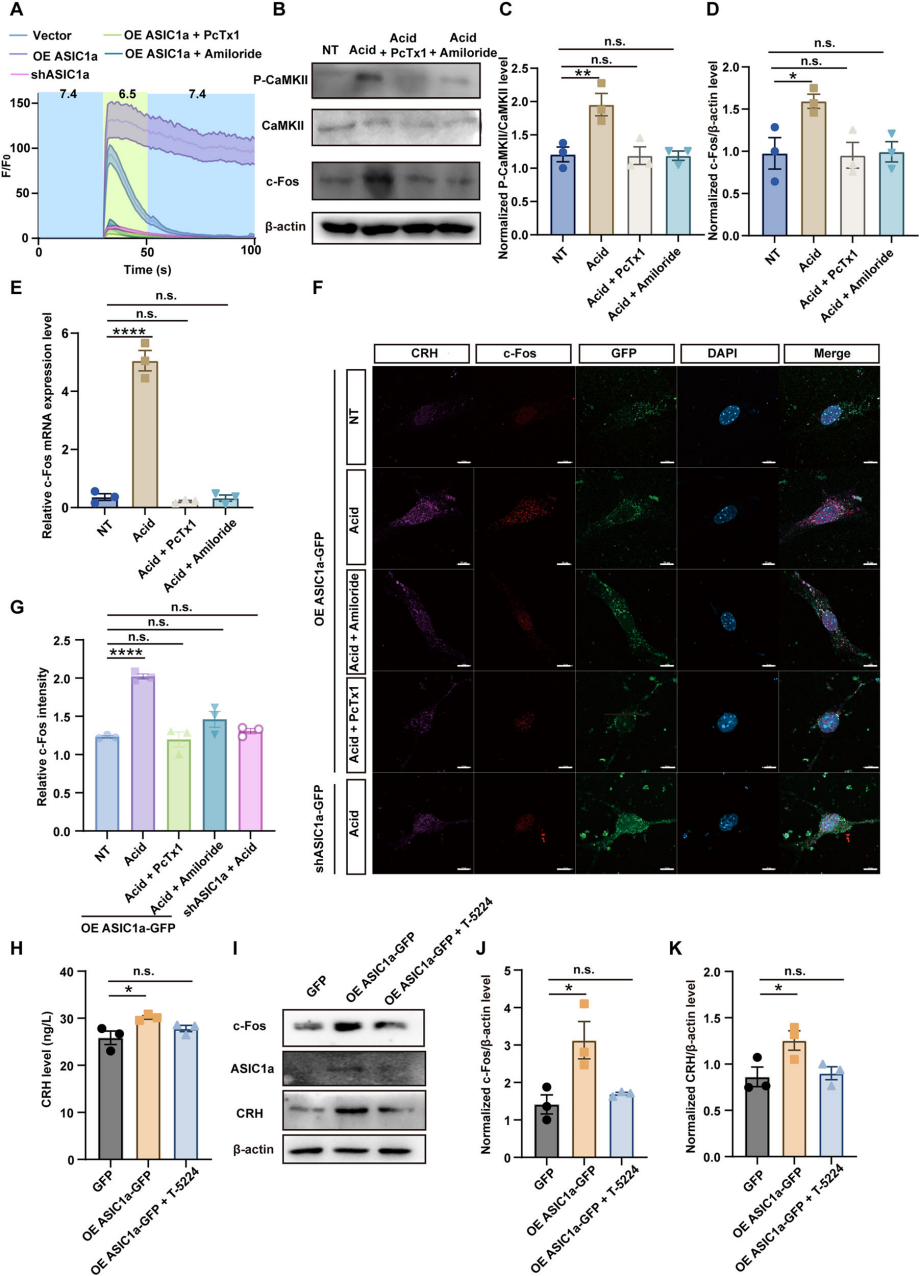
ASIC1a facilitated CRH transcription via Ca^2+^/CaMKII/c-Fos pathway. **A** Acid (pH 6.5)-induced changes in cytosolic Ca^2+^ signal, indicated by GCaMP6 fluorescence, in cultured mouse hypothalamic neurons (n = 77 in Vector group, n = 63 in OE ASIC1a group, n = 48 in shASIC1a group, n = 73 in OE ASIC1a + PcTx1 group, n = 60 in OE ASIC1a + Amiloride group). **B** Representative western blot assay showing the expression of calcium signaling pathway-related proteins in primary hypothalamic neurons. Experimental treatments were conducted as in Fig. 4F. **C** The bar graph illustrates the grayscale scanning analysis of Fig. 5B, showing the relative expression levels of P-CaMKII (n = 3 per group). **p < 0.01 (one-way ANOVA). **D** The bar graph illustrates the grayscale scanning analysis of Fig. 5B, showing the relative expression levels of c-Fos (n = 3 per group). *p < 0.05 (one-way ANOVA). **E** The bar graphs show the mRNA expression levels of c-Fos in primary hypothalamic neurons detected by qPCR. Experimental treatments were conducted as in Fig. 4F (n = 3 per group). ****p < 0.0001 (one-way ANOVA). **F** Representative confocal images (scale bar = 10 μm) of c-Fos and CRH in primary hypothalamic neurons overexpressing GFP/ASIC1-GFP or knockdown of ASIC1a for 48 h, before or 15 min after treatment with HBSS at pH 6.5 with or without 100 nM PcTx1 or 200 nM amiloride. **G** The bar graph illustrated the relative fluorescence intensity of c-Fos shown in Fig. 5F (n = 3 per group). ****p < 0.0001 (one-way ANOVA). **H** The bar graph shows the secretion levels of CRH in primary hypothalamic neuron culture supernatants detected by ELISA, after treatment with pH 6.5 HBSS or simultaneous treatment with 80 μM T-5224 for 30 min following 48-h overexpression of ASIC1a (n = 3 per group). *p < 0.05 (one-way ANOVA). **I** Representative western blot assay showing the protein expression levels of CRH, ASIC1a, and c-Fos in primary hypothalamic neurons. Experimental treatments were conducted as in Fig. 5H. **J** The bar graph illustrates the grayscale scanning analysis of Fig. 5I, showing the relative expression levels of c-Fos (n = 3 per group). *p < 0.05 (one-way ANOVA). **K** The bar graph illustrates the grayscale scanning analysis of Fig. 5I, showing the relative expression levels of CRH (n = 3 per group). *p < 0.05 (one-way ANOVA). See also Supplementary Data 2.

As a Ca^2+^-permeable cation channel, ASIC1a activation was hypothesized to modulate CRH mRNA expression via increasing intracellular Ca^2+^ concentration. We performed Ca^2+^ imaging experiments to show that application of the acidic solution (pH 6.5) elicited a rise in intracellular Ca^2+^ in cultured mouse hypothalamic neurons. This response was enhanced by ASIC1a overexpression and diminished by ASIC1a knockdown or application of the inhibitors PcTx1 and amiloride (Fig. 5A, Supplementary Figure 5A, B). Further analysis revealed that ASIC1a activation significantly influenced the expression of Ca^2+^ signaling-related proteins. Acid stimulation led to a marked elevation in phosphorylated CaMKII levels and a concomitant increase in c-Fos protein levels (Fig. 5B–D). This upregulation of c-Fos occurred at the transcriptional level (Fig. 5E). Given that c-Fos is a critical effector of Ca^2+^ signaling known to regulate CRH expression [45], we confirmed its close association with ASIC1a activation via immunofluorescence (Fig. 5F, G). Activator protein-1 (AP-1) is a complex of c-Fos and c-Jun. The AP-1 inhibitor T-5224, which specifically inhibits the DNA binding activity of AP-1 [46–49]. Since AP-1 is a complex containing c-Fos, it is plausible that T-5224 may also inhibit c-Fos activity. The role of this pathway was functionally confirmed using T-5224, which reduced acid-induced CRH expression and secretion in ASIC1a-overexpressing primary neurons, lowering secretion levels from 30.18 ng/L to 27.82 ng/L, compared to the control’s 25.85 ng/L (Fig. 5H–K).

In mice, we observed a significant increase in phosphorylated CaMKII protein in the PVN of stressed mice compared to the controls (Supplementary Figure 6A, B). To evaluate whether c-Fos modulates the stress response, T-5224 was bilaterally infused into the PVN through implanted catheters in naïve mice, followed by behavioral assessments. The T-5224 group spent more time in the center of the OFT and in the open arms of the EPM compared to the ACSF group (Supplementary Figure 6C, D) and exhibited significant reduction in immobility time in the TST and FST (Supplementary Figure 6E, F). These findings demonstrated that ASIC1a activation promotes CRH transcription by mediating acid-induced calcium influx, which triggers the CaMKII/c-Fos pathway both in vitro and in vivo and subsequently contributes to anxiety- and depressive-like behaviors.

## Discussion

**Fig. 6:**
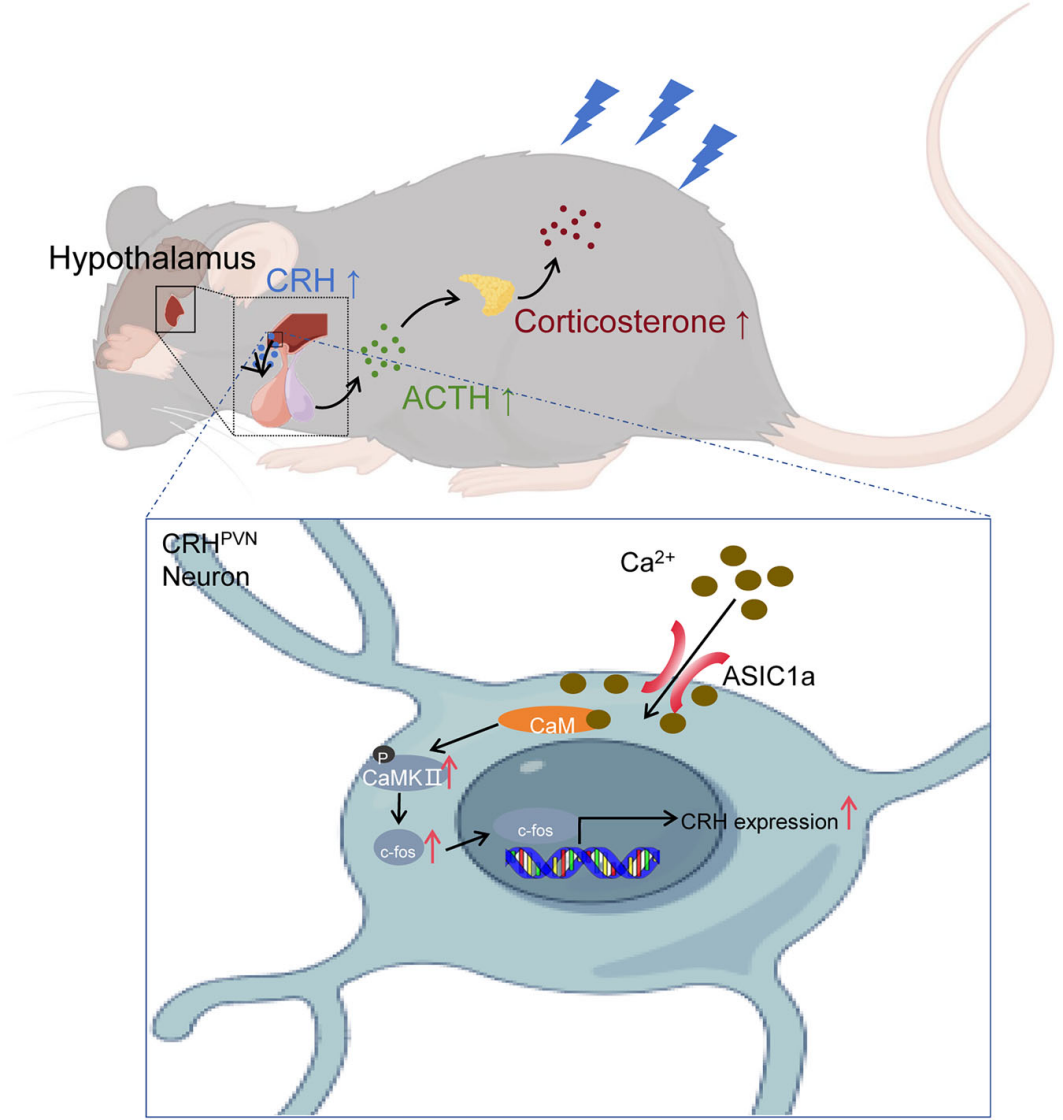
Schematic showing ASIC1a promoted the expression of CRH by activating Ca^2+^/CaMKII/c-Fos signaling pathways. This figure was created by Figdraw (ID: TSRYP14a5b).

This study identified the presence of ASIC1a specifically in the CRH-producing neurons of the paraventricular nucleus (CRH^PVN^ neurons). Using both pharmacological inhibition of ASIC1a in the PVN and AAV-mediated manipulation of ASIC1a expression in CRH^PVN^ neuron, we delineated a prominent influence of ASIC1a on intracellular Ca^2+^ concentration and the activity of CRH^PVN^ neuron, thereby regulating plasma ACTH and corticosterone levels. Down-regulating ASIC1a has been shown to mitigate anxiety- and depression-related behaviors in mice. By modulating ASIC1a expression and employing ASIC1a inhibitors, in conjunction with T-5224 to obstruct the AP-1 DNA binding activity, our evidence solidifies the notion that ASIC1a affects CRH transcription via the Ca^2+^/CaMKII/c-Fos pathway, and activation of ASIC1a promotes CRH release (Fig. 6). Our data indicate that c-Fos is a critical mediator of the acute stress response. While the effects of T-5224 are mediated through AP-1 inhibition, the behavioral and molecular phenotypes we observed are consistent with the blockade of a c-Fos-centric transcriptional program, giving its status as the primary and rapidly inducible component of AP-1 in our model of acute stress.

The CRH^PVN^ neurons serve as a crucial hub for modulation of HPA axis activity and are viewed as the canonical endocrine controller in the modulation of stress [8]. These neurons receive inputs from many brain regions that convey various homeostatic or stress modes to activate the HPA axis upon stress challenge [7]. Ion channels and receptors determine the activity of the CRH^PVN^ neuron, influencing its role in the modulation of stress. For example, glutamatergic transmission is involved in HPA activation [4]. Notably, CRH^PVN^ neurons have recently been demonstrated to orchestrate stress responses independently of endocrine signals, coordinating complex behaviors through excitatory projections to intra-hypothalamus regions such as lateral hypothalamus [10]. CRH^PVN^ neurons also integrate widespread information from extra-hypothalamus regions including forebrain and midbrain through multiple direct long-range GABAergic afferents to modulate stress [8]. The activity of the CRH^PVN^ neuron, is modulated by diverse excitatory and inhibitory inputs from extra- and intra-hypothalamic brain regions. Therefore, the output of the HPA axis is determined by the integration of these signals to modulate stress response. The innate response to predator odor activates CRH neurons in the PVN through the amygdala-piriform transition area and cortical amygdala, which does not require activation of brainstem noradrenergic neurons [50]. Norepinephrine causes a robust activation of glutamatergic synaptic inputs and a less robust activation of GABAergic synaptic inputs to CRH^PVN^ neurons via a postsynaptic α1 adrenoreceptor-dependent retrograde neuronal-glial signaling mechanism, resulting in a net excitation of CRH neurons [9]. Additionally, amygdala excitation will diminish BNST activity and remove the inhibitory tone constraining CRH^PVN^ activation [51]. Newly identifying ion channels and receptors that influencing the CRH^PVN^ neuron activity will no doubt advance our understanding of molecular mechanisms underlying modulations of HPA axis and mood related behaviors.

Manipulating ASIC1a in the PVN affects mood related behaviors, consistent with previous reports on the important involvement of ASIC1a in the modulation of anxiety- and depression-related behaviors [37]. Coryell et al. has demonstrated that downregulation and inhibition of ASIC1a in the amygdala produce antidepressant-like effects [32]. Pharmacological inhibition and genetic knockdown of ASIC1a in the central nucleus of amygdala has shown to ameliorate anxiety-related behaviors [33]. Moreover, ASIC1a increases inhibitory activity in the basolateral amygdala and decreases anxiety during estrus [34]. Also, a crucial role of ASIC1a in the basolateral amygdala in pain and anxiety-related behaviors during arthritis has been reported that blocking ASIC1a in the basolateral amygdala can both reduce pain as well as anxiety related behaviors associated with arthritis [52]. Additionally, targeting ASIC1a gene or acutely inhibiting ASIC1a suppresses fear and anxiety independent of conditioning [27]. Overexpression of ASIC1a in transgenic mice increases fear conditioning [35]. Restoring ASIC1a expression in the basolateral amygdala rescues the CO_2_-induced fear deficit in the ASIC1a null mouse [53]. Our recent study has indicated the presence of ASIC1a in the PVN and inhibition of ASICs may act on the HPA axis to alleviate the depression-related behavior in the chronic stress-subjected rat [37]. Mechanically, our data on immunofluorescence staining in this study further localized the expression of ASIC1a in the CRH^PVN^ neuron. Furthermore, we found that downregulation of ASIC1a in the CRH^PVN^ neuron reduced neuronal activity, alongside the decreased HPA axis activity.

At cellular levels, this study revealed a direct relationship between ASIC1a and CRH expression via Ca^2+^/CaMKII/c-Fos signaling pathway. As an early response gene, CRH is primarily released through de novo synthesis in the PVN in response to stress challenge [54]. The Ca^2+^ signaling pathway is an integral part of the cellular early response system, capable of being activated within seconds to mins to respond to various stimuli [55]. The ASIC1a-induced alteration in intracellular Ca^2+^ concentration impacted CRH neuronal activity, leading to a swift upregulation of CRH mRNA levels. Previous study has shown that CRH promoter harbors AP-1 binding sites, responsive to the c-Fos/AP-1 signaling pathway [56]. This aligns with earlier findings that have observed the co-localization and co-activation of c-Fos and CRH in the PVN [57], along with stress-induced alterations in the expression of both c-Fos and CRH in CRH^PVN^ neuron [58]. Following acid stimulation, we noted a concurrent upregulation of p-CaMKII, and c-Fos in primary hypothalamic neurons. Moreover, T-5224, a selective AP-1 DNA binding blocker was employed to inhibit CRH expression. The rapid activation of CRH neurons in vivo (Fig. 3L) likely reflects ASIC1a-mediated depolarization and immediate calcium influx, which facilitate acute stress responses. In contrast, the slower changes in CRH release and expression observed in vitro (Figs. 4 and 5) may represent downstream effects of prolonged ASIC1a activation under sustained acidic conditions. These findings suggest that ASIC1a contributes to distinct roles across different timescales: mediating rapid neuronal activation and regulating longer-term processes. Therefore, this study elucidates a direct molecular mechanism by which ASIC1a modulates HPA axis and mood related behaviors.

Our preceding study has demonstrated that ASIC1a within the PVN participates in modulating glucose metabolism and energy balance [36]. HPA axis is the major neuroendocrine axis that maintains homeostasis in mammals, including glucose metabolism and energy balance [59]. This study reported that ASIC1a in the PVN modulates the HPA axis activity, which further ameliorated anxiety- and depression-related behaviors by modulating CRH^PVN^ neuronal activity. Some drugs that act on known excitatory neurotransmitter-activated receptor, such as NMDA receptor, have been used to prevent the consolidation of fear memory in rodents. However, drug adverse reactions make them less usable. This study on ASIC1a facilitation of CRH^PVN^ neuronal activity suggests that intervention of ASIC1a could normalize HPA axis function with a potentially more favorable side-effect profile, warranting further exploration. Acetazolamide, a non-selective ASIC1a blocker, is known to inhibit fear conditioning, which strongly supports a tonic, physiologically relevant role for ASIC1a in preventing fear memory consolidation, independent of NMDA receptor function [60]. Therefore, this study could provide a new explanation for the drug treatment. ASIC1a has been extensively implicated in regulating synaptic function, neuronal plasticity and complex behaviors such as learning, memory, and fear responses [41, 61–63]. Especially, ASIC1a has been proven to contribute to both major forms of synaptic plasticity: long-term potentiation (LTP) and long-term depression (LTD) in different physiological and pathological conditions. For example, in the striatum, ASIC1a enhances excitatory synaptic function, thereby supporting striatum-dependent plasticity as well as procedural learning and memory [20]. The evidence from the hippocampal-prefrontal circuitry study indicates that ASIC1a contributes to extinction-driven plasticity via modulation of NMDA receptor activity [64]. Previous studies have confirmed that ASIC1a is activated by extracellular protons due to synaptic vesicle release during neurotransmission and contributes to excitatory postsynaptic currents [31, 65, 66]. A study by Coryell and work colleagues has revealed that ASIC1a in the amygdala is activated by endogenous pH fluctuations during emotional stress [32]. These studies suggest ASIC1a in the PVN might be activated by local acidification in response to acute stress.

Some limitations should be noted in this study. Firstly, this study mainly focuses on the immediate effects of ASIC1a on acute stress responses, leaving a gap in understanding the role of ASIC1a in long-term stress impacts on stress-related behaviors and diseases. Secondly, despite delving into how ASIC1a facilitates CRH transcription through the Ca^2+^/CaMKII/c-Fos pathway, other signaling pathways should be existed and are needed to be investigated. Thirdly, we inferred that ASIC1a was activated under stress conditions without direct measurement of in vivo micro-pH dynamics in the PVN. Finally, the study was conducted only in male mice in order to avoid the estrus cycle-induced variation. Further study in female mice should be carried out to explore whether ASIC1a have different physiologies depending on gender.

Collectively, this study presents a novel modulation mechanism of HPA axis and mood behaviors by ASIC1a in the CRH^PVN^ neuron through ASIC1a-mediated intracellular Ca^2+^ signaling pathway. In the condition of acute stress, increased activity of CRH^PVN^ neuron causes activation of HPA axis activity through ASIC1a-mediated elevated CRH expression that is partly attributed to ASIC1a/Ca^2+^/CaMKII/c-Fos signaling pathway. Therefore, this study proposes ASIC1a as a potential therapeutic target for stress-related diseases such as depression, anxiety and diabetes.

## Supplementary information

Supplementary Figure

Supplementary Data 1

Supplementary Data2

## Data Availability

Data will be made available on request.

## References

[1] Luo YJ, Bao H, Crowther A, Li YD, Chen ZK, Tart DS, et al. Sex-specific expression of distinct serotonin receptors mediates stress vulnerability of adult hippocampal neural stem cells in mice. Cell Rep. 2024;43:114140. 38656873 10.1016/j.celrep.2024.114140 PMC11193935

[2] Agorastos A, Chrousos GP. The neuroendocrinology of stress: the stress-related continuum of chronic disease development. Mol Psychiatry. 2022;27:502–13. 34290370 10.1038/s41380-021-01224-9

[3] Häusl AS, Brix LM, Hartmann J, Pöhlmann ML, Lopez JP, Menegaz D, et al. The co-chaperone Fkbp5 shapes the acute stress response in the paraventricular nucleus of the hypothalamus of male mice. Mol Psychiatry. 2021;26:3060–76. 33649453 10.1038/s41380-021-01044-x PMC8505251

[4] Daviu N, Füzesi T, Rosenegger DG, Rasiah NP, Sterley TL, Peringod G, et al. Paraventricular nucleus CRH neurons encode stress controllability and regulate defensive behavior selection. Nat Neurosci. 2020;23:398–410. 32066984 10.1038/s41593-020-0591-0

[5] Roth MK, Bingham B, Shah A, Joshi A, Frazer A, Strong R, et al. Effects of chronic plus acute prolonged stress on measures of coping style, anxiety, and evoked HPA-axis reactivity. Neuropharmacology. 2012;63:1118–26. 22842072 10.1016/j.neuropharm.2012.07.034 PMC3427462

[6] Herman JP, McKlveen JM, Ghosal S, Kopp B, Wulsin A, Makinson R, et al. Regulation of the hypothalamic-pituitary-adrenocortical stress response. Compr Physiol. 2016;6:603–21. 27065163 10.1002/cphy.c150015 PMC4867107

[7] Yu J, Li XF, Tsaneva-Atanasova K, Zavala E, O’Byrne KT. Chemogenetic activation of PVN CRH neurons disrupts the estrous cycle and LH dynamics in female mice. Front Endocrinol (Lausanne). 2023;14:1322662. 38264285 10.3389/fendo.2023.1322662 PMC10803550

[8] Yuan Y, Wu W, Chen M, Cai F, Fan C, Shen W, et al. Reward inhibits paraventricular CRH neurons to relieve stress. Curr Biol. 2019;29:1243–51.e4. 30853436 10.1016/j.cub.2019.02.048

[9] Jiang Z, Chen C, Weiss GL, Fu X, Stelly CE, Sweeten BLW, et al. Stress-induced glucocorticoid desensitizes adrenoreceptors to gate the neuroendocrine response to somatic stress in male mice. Cell Rep. 2022;41:111509. 36261014 10.1016/j.celrep.2022.111509 PMC9635929

[10] Füzesi T, Daviu N, Wamsteeker Cusulin JI, Bonin RP, Bains JS. Hypothalamic CRH neurons orchestrate complex behaviours after stress. Nat Commun. 2016;7:11937. 27306314 10.1038/ncomms11937 PMC4912635

[11] Füzesi T, Rasiah NP, Rosenegger DG, Rojas-Carvajal M, Chomiak T, Daviu N, et al. Hypothalamic CRH neurons represent physiological memory of positive and negative experience. Nat Commun. 2023;14:8522. 38129411 10.1038/s41467-023-44163-5 PMC10739955

[12] Zhou JJ, Gao Y, Zhang X, Kosten TA, Li DP. Enhanced hypothalamic NMDA receptor activity contributes to hyperactivity of HPA axis in chronic stress in male rats. Endocrinology. 2018;159:1537–46. 29390057 10.1210/en.2017-03176 PMC5839733

[13] Yu L, Zhu X, Peng K, Qin H, Yang K, Cai F, et al. Propofol alleviates anxiety-like behaviors associated with pain by inhibiting the hyperactivity of PVN(CRH) neurons via GABA(A) receptor β3 subunits. Adv Sci (Weinh). 2024; 10.1002/advs.202309059e2309059. 10.1002/advs.202309059 PMC11267288 38639389

[14] Blair LJ, Criado-Marrero M, Zheng D, Wang X, Kamath S, Nordhues BA, et al. The disease-associated chaperone FKBP51 impairs cognitive function by accelerating AMPA receptor recycling. eNeuro. 2019;6:ENEURO.0242-18.2019. 30963102 10.1523/ENEURO.0242-18.2019 PMC6450497

[15] Power EM, Iremonger KJ. Plasticity of intrinsic excitability across the estrous cycle in hypothalamic CRH neurons. Sci Rep. 2021;11:16700. 34404890 10.1038/s41598-021-96341-4 PMC8371084

[16] Zhou JJ, Gao Y, Kosten TA, Zhao Z, Li DP. Acute stress diminishes M-current contributing to elevated activity of hypothalamic-pituitary-adrenal axis. Neuropharmacology. 2017;114:67–76. 27908768 10.1016/j.neuropharm.2016.11.024 PMC5183563

[17] Yamada A, Ling J, Yamada AI, Furue H, Gu JG. ASICs mediate fast excitatory synaptic transmission for tactile discrimination. Neuron. 2024;112:1286–301.e8. 38359825 10.1016/j.neuron.2024.01.018 PMC11031316

[18] Nomura K, Hiyama TY, Sakuta H, Matsuda T, Lin CH, Kobayashi K, et al. [Na(+)] increases in body fluids sensed by central Na(x) induce sympathetically mediated blood pressure elevations via H(+)-Dependent activation of ASIC1a. Neuron. 2019;101:60–75.e6. 30503172 10.1016/j.neuron.2018.11.017

[19] Yang J, Qiu L, Strobel M, Kabel A, Zha XM, Chen X. Acid-Sensing ion channels contribute to type III adenylyl cyclase-independent acid sensing of mouse olfactory sensory neurons. Mol Neurobiol. 2020;57:3042–56. 32458389 10.1007/s12035-020-01943-0 PMC7398588

[20] Yu Z, Wu YJ, Wang YZ, Liu DS, Song XL, Jiang Q, et al. The acid-sensing ion channel ASIC1a mediates striatal synapse remodeling and procedural motor learning. Sci Signal. 2018;11:eaar4481. 30087178 10.1126/scisignal.aar4481 PMC6324561

[21] Zha XM, Wemmie JA, Green SH, Welsh MJ. Acid-sensing ion channel 1a is a postsynaptic proton receptor that affects the density of dendritic spines. Proc Natl Acad Sci USA. 2006;103:16556–61. 17060608 10.1073/pnas.0608018103 PMC1621052

[22] Gao J, Duan B, Wang DG, Deng XH, Zhang GY, Xu L, et al. Coupling between NMDA receptor and acid-sensing ion channel contributes to ischemic neuronal death. Neuron. 2005;48:635–46. 16301179 10.1016/j.neuron.2005.10.011

[23] Yang Y, Jin S, Zhang J, Chen W, Lu Y, Chen J, et al. Acid-sensing ion channel 1a exacerbates renal ischemia-reperfusion injury through the NF-κB/NLRP3 inflammasome pathway. J Mol Med (Berl). 2023;101:877–90. 37246982 10.1007/s00109-023-02330-7 PMC10300185

[24] Zhang L, Leng TD, Yang T, Li J, Xiong ZG. Protein Kinase C regulates ASIC1a protein expression and channel function via NF-kB signaling pathway. Mol Neurobiol. 2020;57:4754–66. 32783140 10.1007/s12035-020-02056-4

[25] Zhou RP, Dai BB, Xie YY, Wu XS, Wang ZS, Li Y, et al. Interleukin-1β and tumor necrosis factor-α augment acidosis-induced rat articular chondrocyte apoptosis via nuclear factor-kappaB-dependent upregulation of ASIC1a channel. Biochim Biophys Acta Mol Basis Dis. 2018;1864:162–77. 28986307 10.1016/j.bbadis.2017.10.004

[26] Yang L, Hu X, Mo YY. Acidosis promotes tumorigenesis by activating AKT/NF-κB signaling. Cancer Metastasis Rev. 2019;38:179–88. 30729352 10.1007/s10555-019-09785-6

[27] Coryell MW, Ziemann AE, Westmoreland PJ, Haenfler JM, Kurjakovic Z, Zha XM, et al. Targeting ASIC1a reduces innate fear and alters neuronal activity in the fear circuit. Biol Psychiatry. 2007;62:1140–8. 17662962 10.1016/j.biopsych.2007.05.008

[28] Liu J, Conde K, Zhang P, Lilascharoen V, Xu Z, Lim BK, et al. Enhanced AMPA receptor trafficking mediates the anorexigenic effect of endogenous glucagon-like Peptide-1 in the paraventricular hypothalamus. Neuron. 2017;96:897–909.e5. 29056294 10.1016/j.neuron.2017.09.042 PMC5729931

[29] Agarwal A, Halvorson LM, Legradi G. Pituitary adenylate cyclase-activating polypeptide (PACAP) mimics neuroendocrine and behavioral manifestations of stress: Evidence for PKA-mediated expression of the corticotropin-releasing hormone (CRH) gene. Brain Res Mol Brain Res. 2005;138:45–57. 15882914 10.1016/j.molbrainres.2005.03.016 PMC1950324

[30] Di T, Chen P, Yuan Z, Wang Y, Sha S, Chen L. Dorsal hypothalamic dopaminergic neurons play an inhibitory role in the hypothalamic-pituitary-adrenal axis via activation of D2R in mice. Acta Physiol (Oxf). 2019;225:e13187. 30204307 10.1111/apha.13187

[31] Storozhuk M, Cherninskyi A, Maximyuk O, Isaev D, Krishtal O. Acid-Sensing ion channels: Focus on physiological and some pathological roles in the brain. Curr Neuropharmacol. 2021;19:1570–89. 33550975 10.2174/1570159X19666210125151824 PMC8762183

[32] Coryell MW, Wunsch AM, Haenfler JM, Allen JE, Schnizler M, Ziemann AE, et al. Acid-sensing ion channel-1a in the amygdala, a novel therapeutic target in depression-related behavior. J Neurosci. 2009;29:5381–8. 19403806 10.1523/JNEUROSCI.0360-09.2009 PMC2710967

[33] Shi P, Zhang MJ, Liu A, Yang CL, Yue JY, Hu R, et al. Acid-sensing ion channel 1a in the central nucleus of the amygdala regulates anxiety-like behaviors in a mouse model of acute pain. Front Mol Neurosci. 2022;15:1006125. 36710934 10.3389/fnmol.2022.1006125 PMC9879607

[34] Pidoplichko VI, Aroniadou-Anderjaska V, Figueiredo TH, Wilbraham C, Braga MFM. Increased inhibitory activity in the basolateral amygdala and decreased anxiety during estrus: A potential role for ASIC1a channels. Brain Res. 2021;1770:147628. 34454948 10.1016/j.brainres.2021.147628

[35] Wemmie JA, Coryell MW, Askwith CC, Lamani E, Leonard AS, Sigmund CD, et al. Overexpression of acid-sensing ion channel 1a in transgenic mice increases acquired fear-related behavior. Proc Natl Acad Sci USA. 2004;101:3621–6. 14988500 10.1073/pnas.0308753101 PMC373512

[36] Wang W, Xu M, Yue J, Zhang Q, Nie X, Jin Y, et al. Knockdown of Acid-sensing ion channel 1a in the PVN promotes metabolic disturbances in male mice. Endocrinology. 2022;163:bqac115. 35894166 10.1210/endocr/bqac115

[37] Zhou W, Ye S, Luo R, Wu LM, Wang W. Inhibition of acid-sensing ion channels reduces the hypothalamus-pituitary-adrenal axis activity and ameliorates depression-like behavior in rats. RSC Adv. 2019;9:8707–13. 35517700 10.1039/c9ra00020h PMC9061884

[38] Cheng Y, Zhang Q, Meng Q, Xia T, Huang Z, Wang C, et al. Leucine deprivation stimulates fat loss via increasing CRH expression in the hypothalamus and activating the sympathetic nervous system. Mol Endocrinol. 2011;25:1624–35. 21719534 10.1210/me.2011-0028 PMC3165911

[39] Zhigulin AS, Tikhonov DB, Barygin OI. Mechanisms of acid-sensing ion channels inhibition by nafamostat, sepimostat and diminazene. Eur J Pharmacol. 2023;938:175394. 36403685 10.1016/j.ejphar.2022.175394

[40] Gitterman DP, Wilson J, Randall AD. Functional properties and pharmacological inhibition of ASIC channels in the human SJ-RH30 skeletal muscle cell line. J Physiol. 2005;562:759–69. 15576453 10.1113/jphysiol.2004.075069 PMC1665529

[41] Wemmie JA, Chen J, Askwith CC, Hruska-Hageman AM, Price MP, Nolan BC, et al. The acid-activated ion channel ASIC contributes to synaptic plasticity, learning, and memory. Neuron. 2002;34:463–77. 11988176 10.1016/s0896-6273(02)00661-x

[42] Yang ZJ, Ni X, Carter EL, Kibler K, Martin LJ, Koehler RC. Neuroprotective effect of acid-sensing ion channel inhibitor psalmotoxin-1 after hypoxia-ischemia in newborn piglet striatum. Neurobiol Dis. 2011;43:446–54. 21558004 10.1016/j.nbd.2011.04.018 PMC3116689

[43] Pignataro G, Simon RP, Xiong ZG. Prolonged activation of ASIC1a and the time window for neuroprotection in cerebral ischaemia. Brain. 2007;130:151–8. 17114797 10.1093/brain/awl325

[44] Wang YZ, Wang JJ, Huang Y, Liu F, Zeng WZ, Li Y, et al. Tissue acidosis induces neuronal necroptosis via ASIC1a channel independent of its ionic conduction. Elife. 2015;4:e05682. 26523449 10.7554/eLife.05682 PMC4629285

[45] Keller-Wood M. Hypothalamic-Pituitary–Adrenal axis-feedback control. Compr Physiol. 2015;5:1161–82. 26140713 10.1002/cphy.c140065

[46] Su H, Liang L, Wang J, Yuan X, Zhao B. ZFP36, an RNA-binding protein promotes hBMSCs osteogenic differentiation via binding with JUN. J Orthop Surg Res. 2024;19:758. 39543732 10.1186/s13018-024-05232-7 PMC11562521

[47] Aikawa Y, Morimoto K, Yamamoto T, Chaki H, Hashiramoto A, Narita H, et al. Treatment of arthritis with a selective inhibitor of c-Fos/activator protein-1. Nat Biotechnol. 2008;26:817–23. 18587386 10.1038/nbt1412

[48] Wang HN, Ji K, Zhang LN, Xie CC, Li WY, Zhao ZF, et al. Inhibition of c-Fos expression attenuates IgE-mediated mast cell activation and allergic inflammation by counteracting an inhibitory AP1/Egr1/IL-4 axis. J Transl Med. 2021;19:261. 34130714 10.1186/s12967-021-02932-0 PMC8207675

[49] Yu W, Gu Y, Zhang H, Huang W, Sun L, Ma L, et al. SF3A3 drives tumorigenesis in endometrial cancer by enhancing c-FOS expression and represents a potential therapeutic target. Adv Sci (Weinh). 2025;12:e04184. 40598817 10.1002/advs.202504184 PMC12463058

[50] Kondoh K, Lu Z, Ye X, Olson DP, Lowell BB, Buck LB. A specific area of olfactory cortex involved in stress hormone responses to predator odours. Nature. 2016;532:103–6. 27001694 10.1038/nature17156 PMC5094457

[51] Rasiah NP, Loewen SP, Bains JS. Windows into stress: a glimpse at emerging roles for CRH(PVN) neurons. Physiol Rev. 2023;103:1667–91. 36395349 10.1152/physrev.00056.2021

[52] Aissouni Y, El Guerrab A, Hamieh AM, Ferrier J, Chalus M, Lemaire D, et al. Acid-Sensing Ion Channel 1a in the amygdala is involved in pain and anxiety-related behaviours associated with arthritis. Sci Rep. 2017;7:43617. 28321113 10.1038/srep43617 PMC5340794

[53] Ziemann AE, Allen JE, Dahdaleh NS, Drebot II, Coryell MW, Wunsch AM, et al. The amygdala is a chemosensor that detects carbon dioxide and acidosis to elicit fear behavior. Cell. 2009;139:1012–21. 19945383 10.1016/j.cell.2009.10.029 PMC2808123

[54] Aguilera G, Liu Y. The molecular physiology of CRH neurons. Front Neuroendocrinol. 2012;33:67–84. 21871477 10.1016/j.yfrne.2011.08.002 PMC4341841

[55] Upadhyaya CP, Gururani MA, Prasad R, Verma A. A cell wall extract from Piriformospora indica promotes tuberization in potato (Solanum tuberosum L.) via enhanced expression of Ca(+2) signaling pathway and lipoxygenase gene. Appl Biochem Biotechnol. 2013;170:743–55. 23609909 10.1007/s12010-013-0231-1

[56] Yoshida M. Gene regulation system of vasopressin and corticotropin-releasing hormone. Gene Regul Syst Bio. 2008;2:71–88. 19787076 10.4137/grsb.s424 PMC2733102

[57] Tanaka T, Shimizu S, Ueno M, Fujihara Y, Ikawa M, Miyata S. MARCKSL1 regulates spine formation in the amygdala and controls the hypothalamic-pituitary-adrenal axis and anxiety-like behaviors. EBioMedicine. 2018;30:62–73. 29580842 10.1016/j.ebiom.2018.03.018 PMC5952351

[58] Wamsteeker Cusulin JI, Füzesi T, Watts AG, Bains JS. Characterization of corticotropin-releasing hormone neurons in the paraventricular nucleus of the hypothalamus of Crh-IRES-Cre mutant mice. PLoS One. 2013;8:e64943. 23724107 10.1371/journal.pone.0064943 PMC3665778

[59] Si MW, Yang MK, Fu XD. Effect of hypothalamic-pituitary-adrenal axis alterations on glucose and lipid metabolism in diabetic rats. Genet Mol Res. 2015;14:9562–70. 26345889 10.4238/2015.August.14.19

[60] Yang MT, Chien WL, Lu DH, Liou HC, Fu WM. Acetazolamide impairs fear memory consolidation in rodents. Neuropharmacology. 2013;67:412–8. 23231808 10.1016/j.neuropharm.2012.11.031

[61] Wemmie JA, Askwith CC, Lamani E, Cassell MD, Freeman JH Jr., Welsh MJ. Acid-sensing ion channel 1 is localized in brain regions with high synaptic density and contributes to fear conditioning. J Neurosci. 2003;23:5496–502. 12843249 10.1523/JNEUROSCI.23-13-05496.2003 PMC6741257

[62] Chu XP, Xiong ZG. Physiological and pathological functions of acid-sensing ion channels in the central nervous system. Curr Drug Targets. 2012;13:263–71. 22204324 10.2174/138945012799201685 PMC3387559

[63] Huang Y, Jiang N, Li J, Ji YH, Xiong ZG, Zha XM. Two aspects of ASIC function: Synaptic plasticity and neuronal injury. Neuropharmacology. 2015;94:42–8. 25582290 10.1016/j.neuropharm.2014.12.010 PMC4458418

[64] Wang Q, Wang Q, Song XL, Jiang Q, Wu YJ, Li Y, et al. Fear extinction requires ASIC1a-dependent regulation of hippocampal-prefrontal correlates. Sci Adv. 2018;4:eaau3075. 30417090 10.1126/sciadv.aau3075 PMC6223961

[65] Du J, Reznikov LR, Price MP, Zha XM, Lu Y, Moninger TO, et al. Protons are a neurotransmitter that regulates synaptic plasticity in the lateral amygdala. Proc Natl Acad Sci USA. 2014;111:8961–6. 24889629 10.1073/pnas.1407018111 PMC4066526

[66] Kreple CJ, Lu Y, Taugher RJ, Schwager-Gutman AL, Du J, Stump M, et al. Acid-sensing ion channels contribute to synaptic transmission and inhibit cocaine-evoked plasticity. Nat Neurosci. 2014;17:1083–91. 24952644 10.1038/nn.3750 PMC4115047

