# Supplementary material for: The acid-sensing ion channel 1a modulates anxiety- and depression-related behaviors via its influencing on the activity of corticotropin-releasing hormone-expressing neurons in the hypothalamic paraventricular nucleus in male mice
Source: Transl Psychiatry. 2026 Mar 19;16:189. doi: 10.1038/s41398-026-03946-2 (PMC13040006; doi:10.1038/s41398-026-03946-2)
Supplement: Supplementary file 1 — Supplementary Figure [file 41398_2026_3946_MOESM1_ESM.docx]

**Supplementary Figure 1**


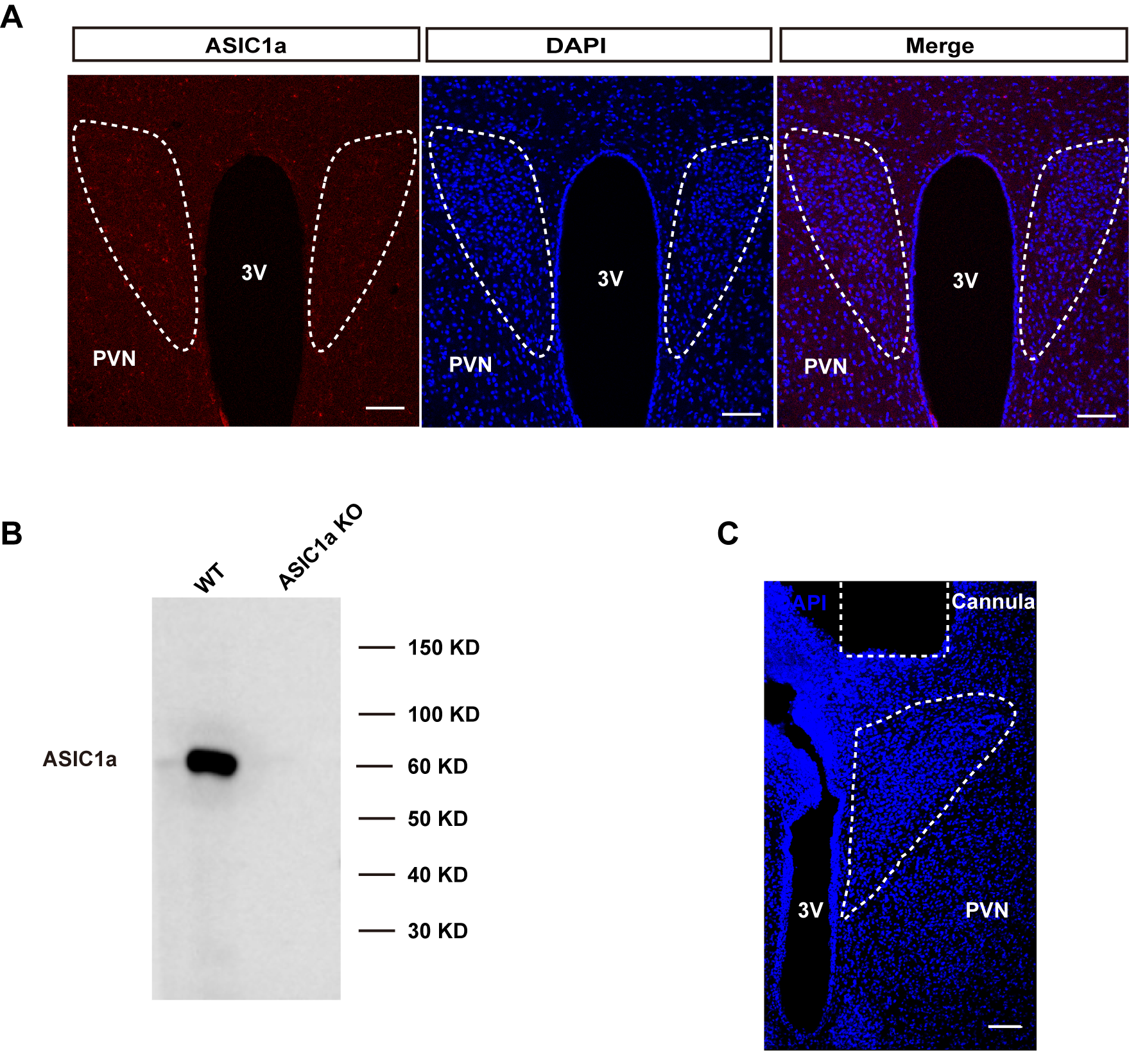


**Supplementary Figure 1**

(A) Representative images of ASIC1a in PVN in the ASIC1a knockout mice. Scale bar, 50 μm.

(B) ASIC1a Western blotting showed a single band of ~60 kD in the C57BL/6J mice (left) and no band in the ASIC1a knockout mice (right).

(C) Representative image of cannula injection site. Scale bar, 100 μm.

**Supplementary Figure 2**


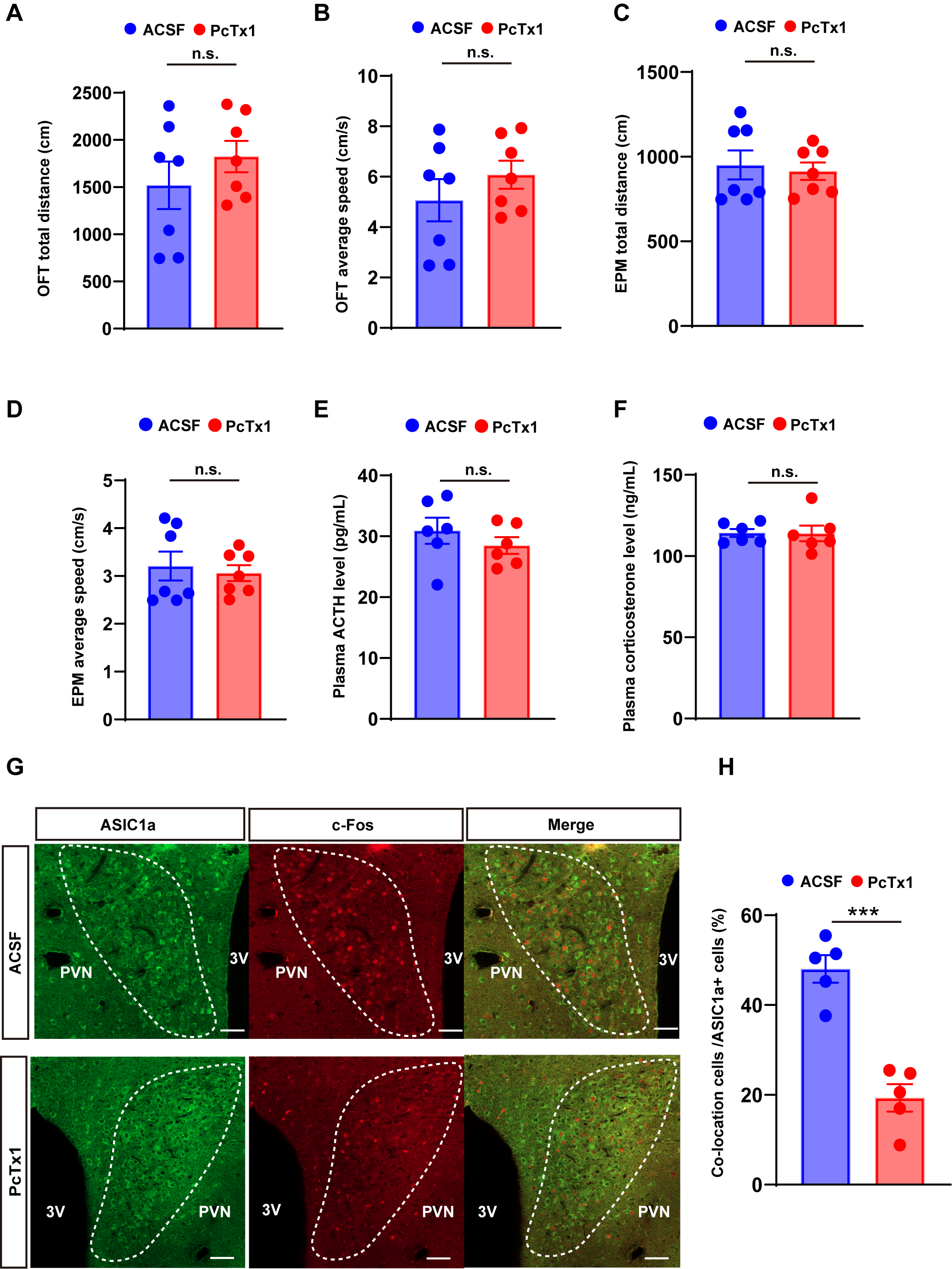


**Supplementary Figure 2**

(A-D) Summarized data showing no difference in total distance traveled (A) and average speed (B) in OFT and no difference in total distance traveled (C) and average speed (D) in EPM between ACSF and PcTx1 groups (n = 7 per group).

(E, F) Baseline plasma ACTH (E) and corticosterone (F) levels prior to stress showed no significant differences between ACSF and PcTx1 groups (n = 6 per group).

(G, H) The representative images (G) and statistics data (H) showed that cannula injection of PcTx1 decreased c-Fos expression within ASIC1a-positive neurons after force swimming in the paraventricular nucleus (PVN) (n = 5 per group). Scale bar, 50 μm. ****p* < 0.001 (unpaired *t*-test). See also Supplementary Data 2.

**Supplementary Figure 3**


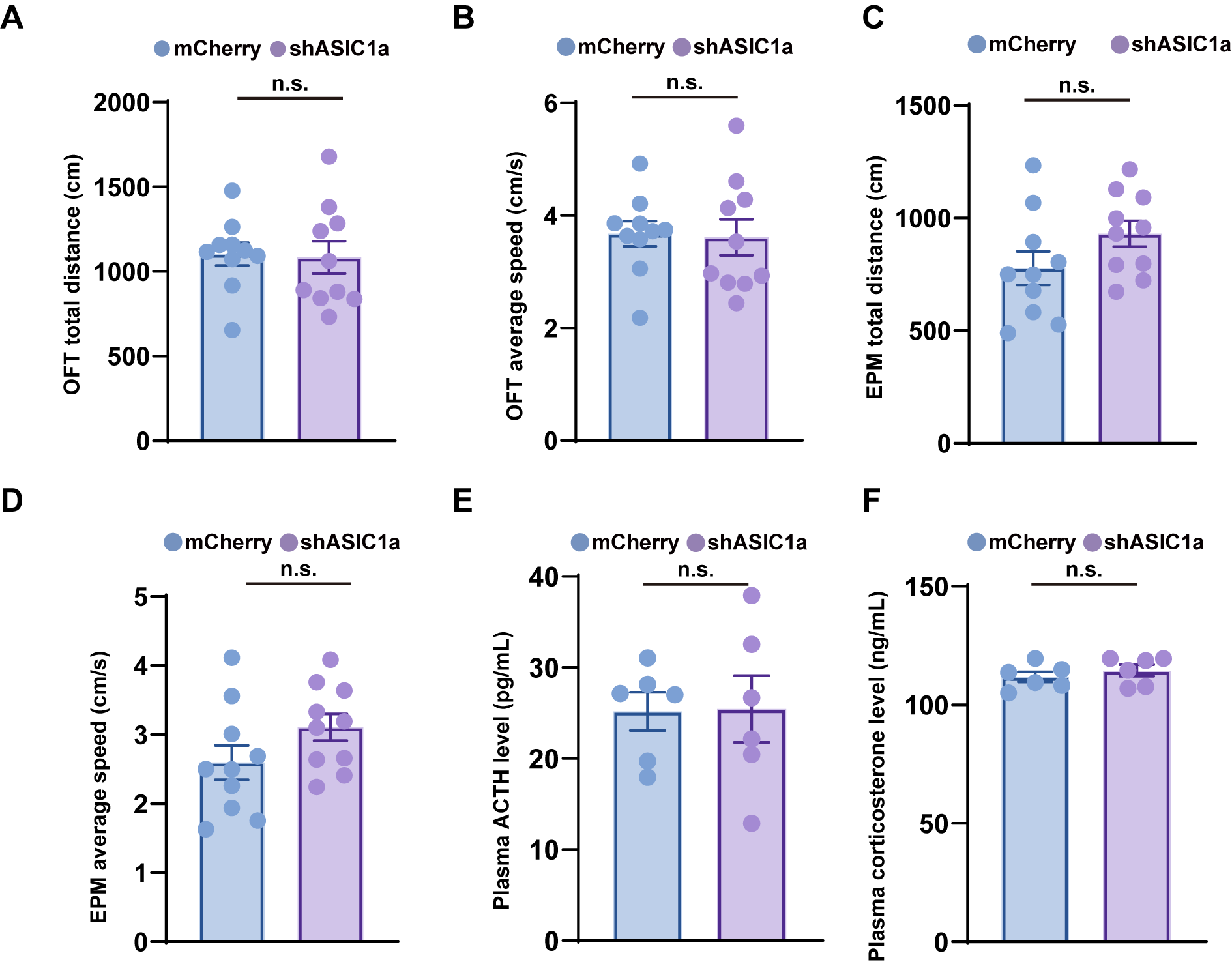


**Supplementary Figure 3**

(A-D) Summarized data showing no difference in total distance traveled (A) and average speed (B) in OFT and no difference in total distance traveled (C) and average speed (D) in EPM between AAV-mCherry and AAV-shASIC1a groups (n = 10 per group).

(E, F) Baseline plasma ACTH (E) and corticosterone (F) levels prior to stress showed no significant differences between AAV-mCherry and AAV-shASIC1a groups (n = 6 per group). See also Supplementary Data 2.

**Supplementary Figure 4**


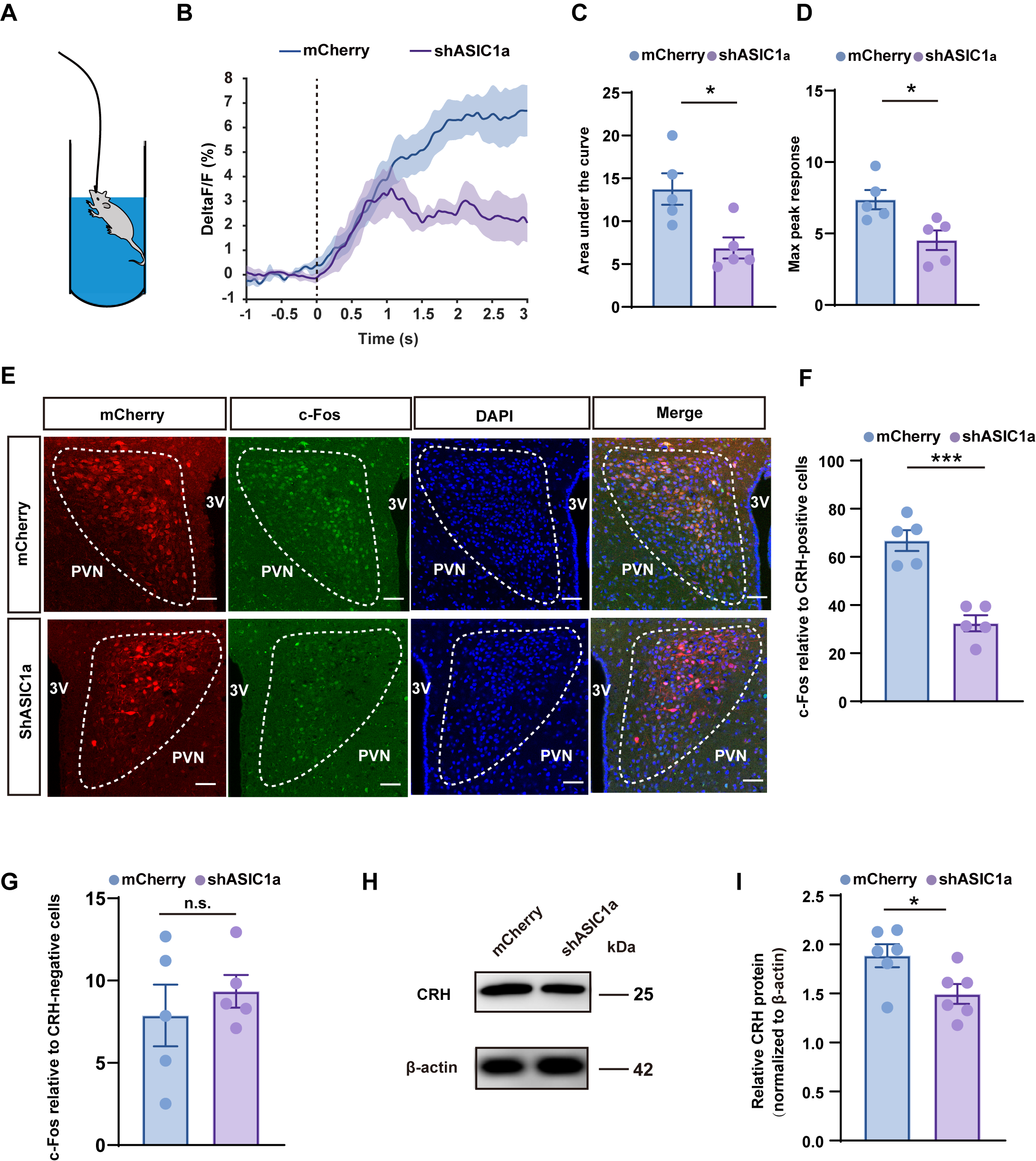


**Supplementary Figure 4**

(A-D) Schematic for FST (A) and calcium activity analysis of CRH^PVN^ neurons in the AAV-shASIC1a group compared to the AAV-mCherry group. Statistics data showed average calcium activity (B), the area under the curve (C), and the maximum peak value (D) (n = 5 per group). **p* < 0.05 (unpaired t-test).

(E-G) The representative images (E) and statistics data showed that Cre-dependent shASIC1a in the PVN of *Crh*-Cre mice decreased c-Fos expression within the mCherry-positive cells (F) and showed no significant change within the mCherry-negative cells (G) after force swimming (n = 5 per group). Scale bar, 50 μm. ****p* < 0.001 (unpaired t-test).

(H, I) Representative Western blot images (H) and quantification (I) demonstrating that AAV-DIO-shASIC1a injection reduced CRH expression in the PVN following exposure to the forced swim test (n = 6 per group). **p* < 0.05 (unpaired t-test). See also Supplementary Data 2.

**Supplementary Figure 5**


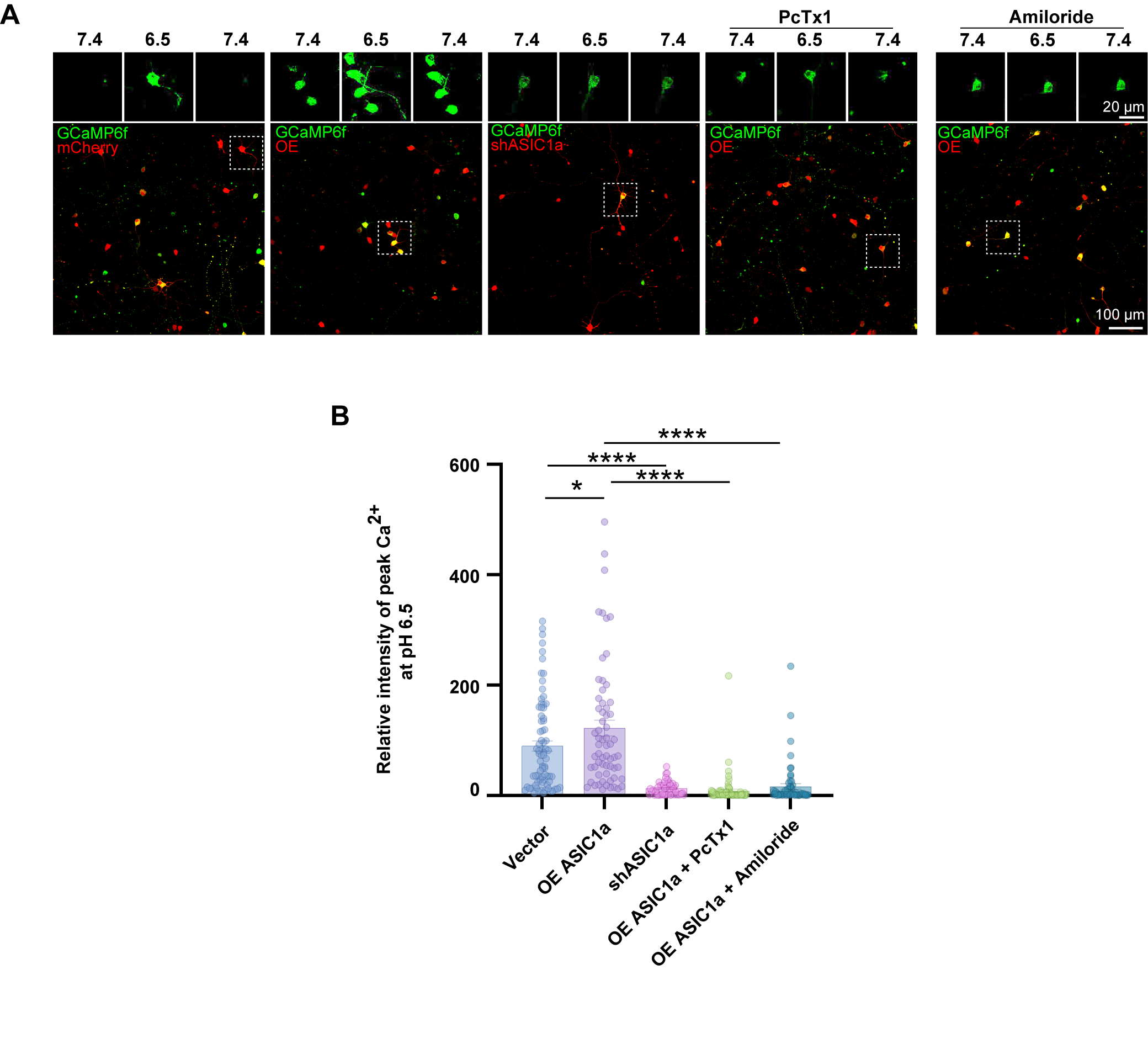


**Supplementary Figure 5**

(A, B) Representative images of GCaMP6f-labeled neurons (A) at indicated time points (7.4, 6.5, 7.4) and statistical analysis of Relative intensity of peak Ca^2+^ at pH 6.5 (B) among five groups. (n = 77 in Vector group, n = 63 in OE ASIC1a group，n = 48 in shASIC1a group, n = 73 in OE ASIC1a + PcTx1 group, n = 60 in OE ASIC1a + Amiloride group). **p* < 0.05, *****p* < 0.0001 (one-way ANOVA). See also Supplementary Data 2.

**Supplementary Figure 6**


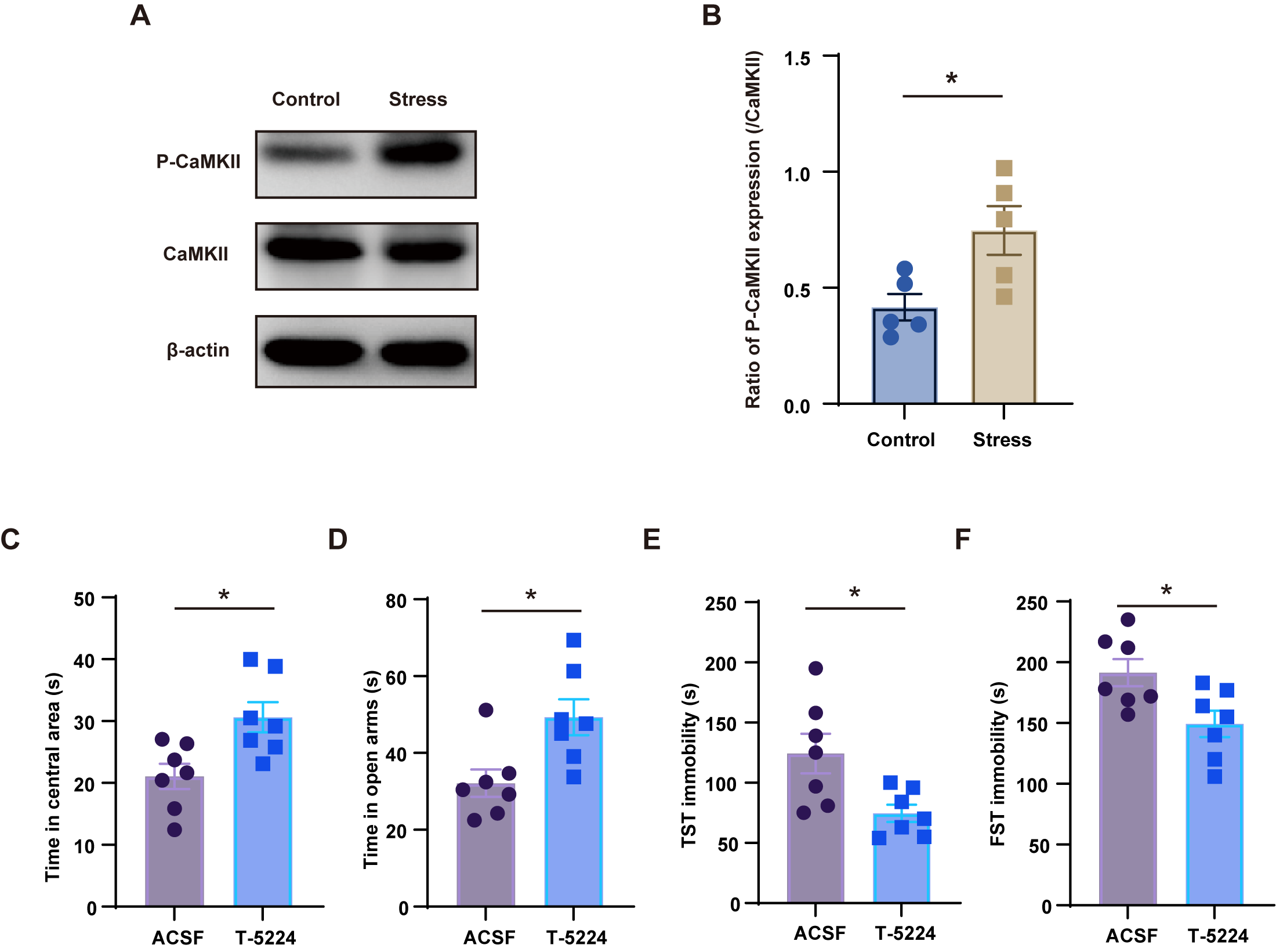


**Supplementary Figure 6**

(A, B) Representative Western blot images (A) and quantification (B) demonstrating that a significant increase in phosphorylated CaMKII protein in the PVN after stress compared to the control (n = 5 per group). **p* < 0.05 (unpaired t-test).

(C) The bar graph illustrates the behavioral analysis, showing that the T-5224 group exhibited a significant increase in the time spent in the center of the open-field test (OFT) compared to the ACSF group (n = 7 per group). **p* < 0.05 (unpaired t-test).

(D) The bar graph illustrates the behavioral analysis, showing that the T-5224 group exhibited a significant increase in the time spent in the open arms of the elevated plus maze (EPM) compared to the ACSF group (n = 7 per group). **p* < 0.05 (unpaired t-test).

(E) The bar graph illustrates the results of the tail suspension test (TST), showing that the T-5224 group exhibited a significant reduction in immobility time compared to the ACSF group (n = 7 per group). **p* < 0.05 (unpaired t-test).

(F) The bar graph illustrates the results of the forced swimming test (FST), showing that the T-5224 group exhibited a significant reduction in immobility time compared to the ACSF group (n = 7 per group). **p* < 0.05 (unpaired t-test). See also Supplementary Data 2.
