# Supplementary material for: The acid-sensing ion channel 1a modulates anxiety- and depression-related behaviors via its influencing on the activity of corticotropin-releasing hormone-expressing neurons in the hypothalamic paraventricular nucleus in male mice
Source: Transl Psychiatry. 2026 Mar 19;16:189. doi: 10.1038/s41398-026-03946-2 (PMC13040006; doi:10.1038/s41398-026-03946-2)
Supplement: Supplementary file 2 — Supplementary Data 1 [file 41398_2026_3946_MOESM2_ESM.pdf]

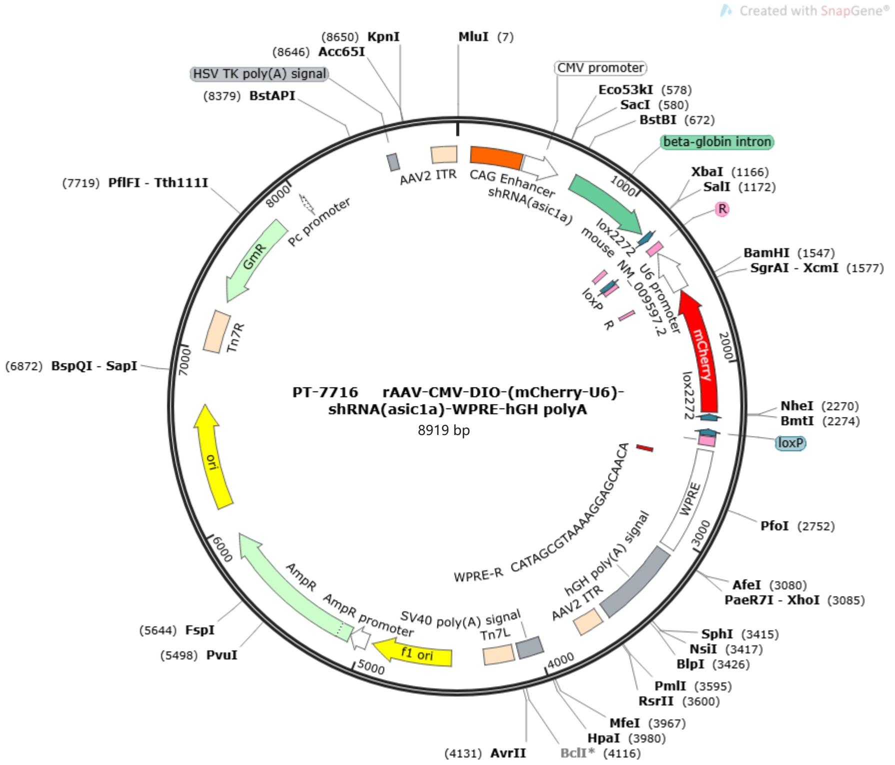

Sequence：

ggccgcacgcgtctagttattaatagtaatcaattacggggtcattagttcatagcccatatatggagttccgcgttacataacttacggtaaat
ggcccgcctggctgaccgcccaacgacccccgcccattgacgtcaataatgacgtatgttcccatagtaacgtcaatagggactttccattga
cgtcaatgggtggagtatttacggtaaactgcccacttggcagtacatcaagtgtatcatatgccaagtacgccccctattgacgtcaatgac
ggtaaatggcccgcctggcattatgcccagtacatgaccttatgggactttcctacttggcagtacatctacgtattagtcatcgctattaccat
ggtgatgcggttttggcagtacatcaatgggcgtggatagcggtttgactcacggggatttccaagtctccaccccattgacgtcaatgggagt
ttgttttgcaccaaaatcaacgggactttccaaaatgtcgtaacaactccgccccattgacgcaaatgggcggtaggcgtgtacggtgggag
gtctatataagcagagctcgtttagtgaaccgtcagatcgcctggagacgccatccacgctgttttgacctccatagaagacaccgggaccga
tccagcctccgcggattcgaatcccggccgggaacggtgcattggaacgcggattccccgtgccaagagtgacgtaagtaccgcctatagag
tctataggcccacaaaaaatgctttcttcttttaatatacttttttgtttatcttatttctaatactttccctaatctctttctttcagggcaataatga
tacaatgtatcatgcctctttgcaccattctaaagaataacagtgataatttctgggttaaggcaatagcaatatttctgcatataaatatttctg
catataaattgtaactgatgtaagaggtttcatattgctaatagcagctacaatccagctaccattctgcttttattttatggttgggataaggct
ggattattctgagtccaagctaggcccttttgctaatcatgttcatacctcttatcttcctcccacagctcctgggcaacgtgctggtctgtgtgct
ggcccatcactttggcaaagaattgggattctagagtcgactccggaataacttcgtataggatactttatacgaagttatgcagaatggtag
ctggattgtagctgctattagcaatatgaaacctcttaataacttcgtatagcatacattatacgaagttattttcgtcctttccacaagatatat
aaagccaagaaatcgaaatactttcaagttacggtaagcatatgatagtccattttaaaacataattttaaaactgcaaactacccaagaaat
tattactttctacgtcacgtattttgtactaatatctttgtgtttacagtcaaattaattccaattatctctctaacagccttgtatcgtatatgcaaa
tatgaaggaatcatgggaaataggccctcGGATCCttacttgtacagctcgtccatgccgccggtggagtggcggccctcggcgcgttcgt
actgttccacgatggtgtagtcctcgttgtgggaggtgatgtccaacttgatgttgacgttgtaggcgccgggcagctgcacgggcttcttggc
cttgtaggtggtcttgacctcagcgtcgtagtggccgccgtccttcagcttcagcctctgcttgatctcgcccttcagggcgccgtcctcggggt
acatccgctcggaggaggcctcccagcccatggtcttcttctgcattacggggccgtcggaggggaagttggtgccgcgcagcttcaccttgt
agatgaactcgccgtcctgcagggaggagtcctgggtcacggtcaccacgccgccgtcctcgaagttcatcacgcgctcccacttgaagccc
tcggggaaggacagcttcaagtagtcggggatgtcggcggggtgcttcacgtaggccttggagccgtacatgaactgaggggacaggatgt
cccaggcgaagggcagggggccaccc ttggtcaccttcagcttggcggtctgggtgccctcgtaggggcggccctcgccctcgccctcgatc
tcgaactcgtggccgttcacggagccctccatgtgcaccttgaagcgcatgaactccttgatgatggccatgttatactcctcgcccttgctcac

catggtggcgctagcataacttcgtataaagtatcctatacgaagttatttgccttaacccagaaattatcactgttattctttagaatggtgca
aagaataacttcgtataatgtatgctatacgaagttatCACCggGGACATTCAGCAAGATGAATATTCAAGAGATATTCATC
TTGCTGAATGTCCTTTTTaattcgatatcaagcttatcgataatcaacctctggattacaaaatttgtgaaagattgactggtattcttaa
ctatgttgctccttttacgctatgtggatacgctgctttaatgcctttgtatcatgctattgcttcccgtatggctttcattttctcctccttgtataaa
tcctggttgctgtctctttatgaggagttgtggcccgttgtcaggcaacgtggcgtggtgtgcactgtgtttgctgacgcaacccccactggttg
gggcattgccaccacctgtcagctcctttccgggactttcgctttccccctccctattgccacggcggaactcatcgccgcctgccttgcccgct
gctggacaggggctcggctgttgggcactgacaattccgtggtgttgtcggggaaatcatcgtcctttccttggctgctcgcctatgttgccacc
tggattctgcgcgggacgtccttctgctacgtcccttcggccctcaatccagcggaccttccttcccgcggcctgctgccggctctgcggcctct
tccgcgtcttcgccttcgccctcagacgagtcggatctccctttgggccgcctccccgcatcgataccgagcgctgctcgagagatctacgggt
ggcatccctgtgacccctccccagtgcctctcctggccctggaagttgccactccagtgcccaccagccttgtcctaataaaattaagttgcat
cattttgtctgactaggtgtccttctataatattatggggtggaggggggtggtatggagcaaggggcaagttgggaagacaacctgtaggg
cctgcggggtctattgggaaccaagctggagtgcagtggcacaatcttggctcactgcaatctccgcctcctgggttcaagcgattctcctgcc
tcagcctcccgagttgttgggattccaggcatgcatgaccaggctcagctaatttttgtttttttggtagagacggggtttcaccatattggcca
ggctggtctccaactcctaatctcaggtgatctacccaccttggcctcccaaattgctgggattacaggcgtgaaccactgctcccttccctgtc
cttctgattttgtaggtaaccacgtgcggaccgagcggccgcaggaacccctagtgatggagttggccactccctctctgcgcgctcgctcgct
cactgaggccgggcgaccaaaggtcgcccgacgcccgggctttgcccgggcggcctcagtgagcgagcgagcgcgcagctgcctgcaggg
gcgcctgatgcggtattttctccttacgcatctgtgcggtatttcacaccgcatacgtcaaagcaaccatagtaactagagcctgcagtctcga
caagcttgtcgagaagtactagaggatcataatcagccataccacatttgtagaggttttacttgctttaaaaaacctcccacacctccccctg
aacctgaaacataaaatgaatgcaattgttgttgttaacttgtttattgcagcttataatggttacaaataaagcaatagcatcacaaatttcac
aaataaagcatttttttcactgcattctagttgtggtttgtccaaactcatcaatgtatcttatcatgtctggatctgatcactgcttgagcctagg
agatccgaaccagataagtgaaatctagttccaaactattttgtcatttttaattttcgtattagcttacgacgctacacccagttcccatctattt
tgtcactcttccctaaataatccttaaaaactccatttccacccctcccagttcccaactattttgtccgcccacagcggggcatttttcttcctgt
tatgtttttaatcaaacatcctgccaactccatgtgacaaaccgtcatcttcggctactttttctctgtcacagaatgaaaatttttctgtcatctct
tcgttattaatgtttgtaattgactgaatatcaacgcttatttgcagcctgaatggcgaatgggacgcgccctgtagcggcgcattaagcgcgg
cgggtgtggtggttacgcgcagcgtgaccgctacacttgccagcgccctagcgcccgctcctttcgctttcttcccttcctttctcgccacgttcg
ccggctttccccgtcaagctctaaatcgggggctccctttagggttccgatttagtgctttacggcacctcgaccccaaaaaacttgattaggg
tgatggttcacgtagtgggccatcgccctgatagacggtttttcgccctttgacgttggagtccacgttctttaatagtggactcttgttccaaac
tggaacaacactcaaccctatctcggtctattcttttgatttataagggattttgccgatttcggcctattggttaaaaaatgagctgatttaaca
aaaatttaacgcgaattttaacaaaatattaacgtttacaatttcaggtggcacttttcggggaaatgtgcgcggaacccctatttgtttattttt
ctaaatacattcaaatatgtatccgctcatgagacaataaccctgataaatgcttcaataatattgaaaaaggaagagtatgagtattcaaca
tttccgtgtcgcccttattcccttttttgcggcattttgccttcctgtttttgctcacccagaaacgctggtgaaagtaaaagatgctgaagatcag
ttgggtgcacgagtgggttacatcgaactggatctcaacagcggtaagatccttgagagttttcgccccgaagaacgttttccaatgatgagc
acttttaaagttctgctatgtggcgcggtattatcccgtattgacgccgggcaagagcaactcggtcgccgcatacactattctcagaatgact
tggttgagtactcaccagtcacagaaaagcatcttacggatggcatgacagtaagagaattatgcagtgctgccataaccatgagtgataac
actgcggccaacttacttctgacaacgatcggaggaccgaaggagctaaccgcttttttgcacaacatgggggatcatgtaactcgccttgat
cgttgggaaccggagctgaatgaagccataccaaacgacgagcgtgacaccacgatgcctgtagcaatggcaacaacgttgcgcaaacta
ttaactggcgaactacttactctagcttcccggcaacaattaatagactggatggaggcggataaagttgcaggaccacttctgcgctcggcc
cttccggctggctggtttattgctgataaatctggagccggtgagcgtgggtctcgcggtatcattgcagcactggggccagatggtaagccct
cccgtatcgtagttatctacacgacggggagtcaggcaactatggatgaacgaaatagacagatcgctgagataggtgcctcactgattaag
cattggtaactgtcagaccaagtttactcatatatactttagattgatttaaaacttcatttttaatttaaaaggatctaggtgaagatcctttttg
ataatctcatgaccaaaatcccttaacgtgagttttcgttccactgagcgtcagaccccgtagaaaagatcaaaggatcttcttgagatcctttt
tttctgcgcgtaatctgctgcttgcaaacaaaaaaaccaccgctaccagcggtggtttgtttgccggatcaagagctaccaactctttttccga
aggtaactggcttcagcagagcgcagataccaaatactgtccttctagtgtagccgtagttaggccaccacttcaagaactctgtagcaccgc
ctacatacctcgctctgctaatcctgttaccagtggctgctgccagtggcgataagtcgtgtcttaccgggttggactcaagacgatagttacc

ggataaggcgcagcggtcgggctgaacggggggttcgtgcacacagcccagcttggagcgaacgacctacaccgaactgagatacctaca
gcgtgagcattgagaaagcgccacgcttcccgaagggagaaaggcggacaggtatccggtaagcggcagggtcggaacaggagagcgc
acgagggagcttccagggggaaacgcctggtatctttatagtcctgtcgggtttcgccacctctgacttgagcgtcgatttttgtgatgctcgtc
aggggggcggagcctatggaaaaacgccagcaacgcggcctttttacggttcctggccttttgctggccttttgctcacatgttctttcctgcgt
tatcccctgattctgtggataaccgtattaccgcctttgagtgagctgataccgctcgccgcagccgaacgaccgagcgcagcgagtcagtga
gcgaggaagcggaagagcgcctgatgcggtattttctccttacgcatctgtgcggtatttcacaccgcagaccagccgcgtaacctggcaaa
atcggttacggttgagtaataaatggatgccctgcgtaagcgggtgtgggcggacaataaagtcttaaactgaacaaaatagatctaaacta
tgacaataaagtcttaaactagacagaatagttgtaaactgaaatcagtccagttatgctgtgaaaaagcatactggacttttgttatggcta
aagcaaactcttcattttctgaagtgcaaattgcccgtcgtattaaagaggggcgtggccaagggcatggtaaagactatattcgcggcgttg
tgacaatttaccgaacaactccgcggccgggaagccgatctcggcttgaacgaattgttaggtggcggtacttgggtcgatatcaaagtgcat
cacttcttcccgtatgcccaactttgtatagagagccactgcgggatcgtcaccgtaatctgcttgcacgtagatcacataagcaccaagcgcg
ttggcctcatgcttgaggagattgatgagcgcggtggcaatgccctgcctccggtgctcgccggagactgcgagatcatagatatagatctca
ctacgcggctgctcaaacctgggcagaacgtaagccgcgagagcgccaacaaccgcttcttggtcgaaggcagcaagcgcgatgaatgtct
tactacggagcaagttcccgaggtaatcggagtccggctgatgttgggagtaggtggctacgtctccgaactcacgaccgaaaagatcaag
agcagcccgcatggatttgacttggtcagggccgagcctacatgtgcgaatgatgcccatacttgagccacctaactttgttttagggcgactg
ccctgctgcgtaacatcgttgctgctccataacatcaaacatcgacccacggcgtaacgcgcttgctgcttggatgcccgaggcatagactgt
acaaaaaaacagtcataacaagccatgaaaaccgccactgcgccgttaccaccgctgcgttcggtcaaggttctggaccagttgcgtgagc
gcatacgctacttgcattacagtttacgaaccgaacaggcttatgtcaactgggttcgtgccttcatccgtttccacggtgtgcgtcacccggca
accttgggcagcagcgaagtcgaggcatttctgtcctggctggcgaacgagcgcaaggtttcggtctccacgcatcgtcaggcattggcggc
cttgctgttcttctacggcaaggtgctgtgcacggatctgccctggcttcaggagatcggtagacctcggccgtcgcggcgcttgccggtggtg
ctgaccccggatgaagtggttcgcatcctcggttttctggaaggcgagcatcgtttgttcgcccaggactctagctatagttctagtggttggcc
tacgtacccgtagtggctatggcagggcttgccgccccgacgttggctgcgagccctgggccttcacccgaacttggggggttggggtgggga
aaaggaagaaacgcgggcgtattggtcccaatggggtctcggtggggtatcgacagagtgccagccctgggaccgaaccccgcgtttatga
acaaacgacccaacacccgtgcgttttattctgtctttttattgccgtcatagcgcgggttccttccggtattgtctccttccgtgtttcagttagcc
tcccccatctcccggtacctccggacctctgacttgagcgtcgatttttgtgatgctcgtcaggggggcggagcctatggaaaaacgccagca
acgcggcctttttacggttcctggccttttgctggccttttgctcacatgtcctgcaggcagctgcgcgctcgctcgctcactgaggccgcccgg
gcaaagcccgggcgtcgggcgacctttggtcgcccggcctcagtgagcgagcgagcgcgcagagagggagtggccaactccatcactagg
ggttcctgc
